# Functional Connectivity of the Human Paraventricular Thalamic Nucleus: Insights From High Field Functional MRI

**DOI:** 10.3389/fnint.2021.662293

**Published:** 2021-04-21

**Authors:** Sarah M. Kark, Matthew T. Birnie, Tallie Z. Baram, Michael A. Yassa

**Affiliations:** ^1^Center for the Neurobiology of Learning and Memory, University of California, Irvine, Irvine, CA, United States; ^2^Department of Neurobiology and Behavior, University of California, Irvine, Irvine, CA, United States; ^3^Department of Pediatrics, University of California, Irvine, Irvine, CA, United States; ^4^Department of Anatomy & Neurobiology, University of California, Irvine, Irvine, CA, United States

**Keywords:** paraventricular thalamic nucleus, reward, resting state functional connectivity, brain circuit, neuroimaging

## Abstract

The paraventricular thalamic nucleus (PVT) is a small but highly connected nucleus of the dorsal midline thalamus. The PVT has garnered recent attention as a context-sensitive node within the thalamocortical arousal system that modulates state-dependent motivated behaviors. Once considered related to generalized arousal responses with non-specific impacts on behavior, accumulating evidence bolsters the contemporary view that discrete midline thalamic subnuclei belong to specialized corticolimbic and corticostriatal circuits related to attention, emotions, and cognition. However, the functional connectivity patterns of the human PVT have yet to be mapped. Here, we combined high-quality, high-resolution 7T and 3T resting state MRI data from 121 young adult participants from the Human Connectome Project (HCP) and thalamic subnuclei atlas masks to investigate resting state functional connectivity of the human PVT. The 7T results demonstrated extensive positive functional connectivity with the brainstem, midbrain, ventral and dorsal medial prefrontal cortex (mPFC), anterior and posterior cingulate, ventral striatum, hippocampus, and amygdala. These connections persist upon controlling for functional connectivity of the rest of the thalamus. Whole-brain contrasts provided further evidence that, compared to three nearby midline thalamic subnuclei, functional connectivity of the PVT is strong with the hippocampus, amygdala, ventral and dorsal mPFC, and middle temporal gyrus. These findings suggest that, even during rest, the human PVT is functionally coupled with many regions known to be structurally connected to rodent and non-human primate PVT. Further, cosine similarity analysis results suggested the PVT is integrated into the default mode network (DMN), an intrinsic connectivity network associated with episodic memory and self-referential thought. The current work provides a much-needed foundation for ongoing and future work examining the functional roles of the PVT in humans.

## Introduction

The thalamus (Greek for “inner chamber”) is well known for its role as a sensory and motor signal relay region. However, accumulating evidence of discrete thalamo-limbic, thalamo-striatal, and thalamo-cortical projections, circuits, and functional connectivity suggests the thalamus acts more as an integrator of specific behaviors than as a passive relay station (Groenewegen and Berendse, 1994; Van der Werf et al., 2002; Kirouac, 2015; Hwang et al., 2017; Kumar et al., 2017). Deep within the medial-dorsal thalamus lies a small collection of densely packed cells called the paraventricular thalamic nucleus (PVT). Decades of rodent and non-human primate literature have established the PVT as an important stress and reward-related node of the limbic network (Bubser and Deutch, 1999). Once considered to be related to generalized arousal responses with non-specific impacts on behavior, the PVT has gained recent attention as an experience-sensitive node of the salience system. It is thought to contribute to encoding and retrieval of emotional memories, especially stress, influencing reward-related behaviors (Fenoglio et al., 2006; Kirouac, 2015; Zhu et al., 2018; Barson et al., 2020; McGinty and Otis, 2020). Indeed, the posterior PVT has recently been referred to as both a potential “stress-memory” center of the brain (Bhatnagar and Dallman, 1998; Bhatnagar et al., 2000; Fenoglio et al., 2006; Heydendael et al., 2011) and the whole nucleus as the “traffic light of motivated behaviors” (McGinty and Otis, 2020). The past decade has seen multiple review articles and commentaries describing the heterogeneity of PVT anatomy, diverse neurochemistry, and functions (James and Dayas, 2013; Do-Monte and Kirouac, 2017; Millan et al., 2017; Barson et al., 2020), including a potential role in long-term fear memory (Padilla-Coreano et al., 2012; Penzo et al., 2015; Do-Monte et al., 2016), salience, conflict resolution (Choi and McNally, 2017; Choi et al., 2019), motivated memory, and associated behaviors (Kirouac, 2015; Millan et al., 2017; Zhu et al., 2018; Zhou and Zhu, 2019; Lucantonio et al., 2020). However, given its small size [e.g., ~7 mm^3^ for left paraventricular nucleus (Krauth et al., 2010; Jo et al., 2019)], there is a dearth in our understanding of PVT function in the human brain.

The PVT was first described in rats by Gurdjian (1927) and later characterized in humans by Hassler (1959). In rodents, the PVT receives afferent projections from the amygdala, lateral hypothalamus (LH), nucleus of the solitary tract, periaqueductal gray area (PAG), parabrachial, raphe nucleus, locus coeruleus, and the suprachiasmatic nucleus (Smith and Sidibe, 2003; Vertes et al., 2015). Primary efferent projections target the amygdala (central and basolateral), ventral subiculum of the hippocampus, bed nucleus of the stria terminalis (BNST), nucleus accumbens (NAc), agranular/dysgranular insular cortex, and the infralimbic and prelimbic cortices (Uroz et al., 2004; Kirouac, 2015). Compared to the anterior PVT (aPVT), the posterior PVT (pPVT) shows more prominent projections to the caudate-putamen, more widespread amygdala labeling, and denser labeling following pPVT injections (Vertes and Hoover, 2008). Conversely, the aPVT is more strongly connected to the hippocampus than the pPVT (Moga et al., 1995; Li and Kirouac, 2012). In aggregate, these studies provide a predictive framework for the potential functional connectivity of the human PVT.

Post-mortem anatomical studies in humans have described the PVT as an unmyelinated, ovoid-shaped structure located along the most medial-dorsal portion of the thalamus (Uroz et al., 2004). There are now 3D atlases of thalamic subnuclei in humans based on multiple histological data (Krauth et al., 2010; Jakab et al., 2012). Yet in human neuroimaging, the PVT is typically combined with other midline subnuclei into a medial or medio-dorsal group. Taking this approach, diffusion tensor imaging work has shown that the mediodorsal thalamus is highly connected with the frontal gyri, temporal pole, anterior cingulate, medial temporal lobe (MTL), basal ganglia, and ventral tegmental area (VTA; Lambert et al., 2017). Functionally, recent work has shown that thalamic subnuclei have strong functional connectivity with multiple other subnuclei as well as several cortical functional networks, suggesting the thalamus integrates information within itself as well as across large-scale functional brain networks (Hwang et al., 2017). In that study, the mediodorsal subdivision showed significant connectivity with the default mode network (DMN) and cingulo-opercular/salience network, consistent with prior work that the thalamus is a part of the DMN (Alves et al., 2019).

Recent work in humans demonstrated discrete thalamo-cortical connectivity related to episode memory—showing medial thalamic engagement was selective to the retrieval phase of episodic memory (Pergola et al., 2013). These results are consistent with the role of the PVT in long-term memory but not the learning or encoding phase (Do-Monte et al., 2016). Unlike the basolateral amygdala, which plays an immediate and general Pavlovian associative role in fear and motivated learning, the recruitment of the PVT might serve to coordinate salient *memories* with biologically adaptive responses and homeostatic regulation (Do-Monte et al., 2016). In addition to probable functional coupling with subcortical, MTL, and striatal regions, it is possible that the PVT is functionally linked with distinct macrostructures, such as the DMN or salience networks.

The PVT has been implicated in depressive-, anxiety-, and fear-like behaviors (Li et al., 2010a,b; Zhu et al., 2011; Hsu et al., 2014), suggesting dysfunction of the PVT might contribute to aberrant behavior and mood states (Do-Monte et al., 2016). In humans, major depression and its symptom severity have been linked with increased functional connectivity between the medial thalamus and cortical areas (Brown et al., 2017). In addition to PVT recruitment to natural rewards, the PVT has also be implicated in drug seeking behaviors and addiction (Matzeu et al., 2014; Chisholm et al., 2021).

All of these diverse and important functions of the PVT, position this region as a key brain node. Thus, understanding the functional connectivity of the PVT in the resting state is a prerequisite for future research to test cognitive and emotional functions of the PVT in both healthy individuals and in psychopathology.

To our knowledge, there is no prior work specifically examining resting state functional connectivity of the PVT using high-resolution 7T resting state functional magnetic resonance imaging (rsfMRI). The present study mapped the resting state functional connectivity of the PVT in a large sample of healthy young adults (*n* = 121). We utilized a 3D atlas of the human thalamus that includes a PVT region (Krauth et al., 2010; Jakab et al., 2012) in combination with high-resolution 7T resting state datasets drawn from the publicly available Human Connectome Project (HCP; Smith et al., 2013; Van Essen et al., 2013).

## Materials and Methods

### Participants

We analyzed 7T and 3T rsfMRI data from the publicly available HCP (Smith et al., 2013; Van Essen et al., 2013). The starting sample was the 184 young healthy adults with 7T rsfMRI data. All participants had normal or corrected-to-normal vision. We limited this sample to adults under the age of 36 years, without anatomical anomalies or segmentation problems noted by the HCP (quality control codes A or B) or other known issues noted on the HCP Wiki page^1^ and with a completed Full NIH Toolbox Battery for optional assessment of neurological and behavioral functioning. We further limited our primary analyses to participants with two, 7T rsfMRI scans collected within the same day (*n* = 135, see “Preprocessing and De-Noising” section for details). From this group, 14 additional participants were excluded for having relatively excessive motion compared to the rest of the sample (see “Preprocessing and De-Noising” section for further details). The final sample submitted for the group rsfMRI analyses consisted of 121 adults (73 females) aged 22–35 (10% age 22–25, 50% age 26–30, and 40% age 31–35).

The study was approved by Washington University in the St. Louis’ Human Research Protection Office (IRB #201204036). Written informed consent was obtained from all study participants. No study activities or procedures with human subjects took place at the authors’ institution. The current secondary analysis of the HCP data was deemed exempt from review by the Institutional Review Board of University of California, Irvine. All data were de-identified by HCP before public release and all HCP participants provided written informed consent to study procedures and data sharing outlined by HCP.

### MRI Acquisition

#### Structural MRI

The 3T structural scan (T1 3D MPRAGE, TR = 2,400 ms, TE = 2.14 ms, flip angle = 8 degrees, FOV = 224 mm × 224 mm, 0.7 mm isotropic voxels). See http://protocols.humanconnectome.org/HCP/7T/. The HCP did not collect a 7T structural. Instead, 3T structural data were downsampled to a 1.6 mm resolution for working with the 7T functional data (HCP filename: T1w_restore_1.60.nii.gz).

#### Resting-State fMRI

The HCP 7T rsfMRI data consisted of two, 16-minute functional scans [900 frames per run, 1.6 mm isotropic voxels, TR = 1000 ms, TE = 22.2 ms, flip angle = 45 degrees, FOV = 208 × 208 mm, 85 slices, multi-band factor = 5, image acceleration factor (iPAT) = 2; Smith et al., 2013; Uğurbil et al., 2013]. See http://protocols.humanconnectome.org/HCP/7T/ for further details. We used one scan from each phase encoding acquisition (anterior-to-posterior and posterior-and-anterior) to eliminate any phase-encoding biases. For each participant, we used the HCP meta-data to identity two resting-state scans that were collected on the same day to minimize across-day changes in mental states. Two participants did not have a usable pair of scans collected on the same day, reducing the sample size at this stage to 135 participants.

The HCP 3T rsfMRI consists of two, 15-min resting state scans (1,200 frames per run, 2.0 mm isotropic voxels, TR = 720 ms, TE = 33.1 ms, flip angle = 52 degrees, FOV = 208 × 180 mm, 72 slices, multi-band factor = 8; Smith et al., 2013; Uğurbil et al., 2013). See http://protocols.humanconnectome.org/HCP/3T/imaging-protocols.html. We again selected pairs of scans in opposing phase encoding directions (left-to-right and right-to-left) collected on the same day to eliminate phase-encoding biases and across-day effects.

### MRI Analysis

#### Preprocessing and De-Noising

We utilized the “minimally preprocessed” datasets provided by the HCP 1200 Release (HCP filename: rfMRI*hp2000_clean.nii.gz), which includes gradient-nonlinearity-induced distortion correction, rigid body head motion correction, EPI image distortion correction, co-registration between the fMRI and structural data, normalization to MNI space, high-pass filtering (1/2,000 Hz), and brain masking (Glasser et al., 2013) and independent components analysis (ICA)-based artifact removal of noise components from preprocessed fMRI data (ICA-FIX; Griffanti et al., 2014; Salimi-Khorshidi et al., 2014).

We entered the minimally preprocessed structural images (HCP filenames: T1w_restore.1.60.nii.gz for 7T and T1w_restore_brain.nii.gz for 3T) and ICA-FIX functionals (rfMRI*hp2000_clean.nii.gz files) into separate projects for the 7T and 3T data using the CONN Toolbox Version 19c (Whitfield-Gabrieli and Nieto-Castanon, 2012,^2^; RRID:SCR_009550). In CONN, the data were then segmented (gray matter, white matter, and CSF) and the functional images were spatially smoothed using a 4 mm and 5 mm full width at half maximum (FWHM) smoothing kernels for the 7T and 3T projects, respectively.

The smoothing kernel sizes were chosen based on prior recommendations to select a kernel FWHM that is 2–3 times the functional voxel size (Mikl et al., 2008; Pajula and Tohka, 2014), which approximates the extent of the underlying signal on the cortex (3–5 mm; Hopfinger et al., 2000). Prior thalamic mapping work has shown very little difference between, for example, the habenula connectivity peak results for smoothed compared to unsmoothed maps, except for in the voxels surrounding the habenula (Ely et al., 2019). Since the current work aimed to map PVT FC with other subcortical structures and cortical areas and not intra-thalamic connectivity, we opted to smooth the functional datasets.

Artifact identification was performed using Artifact Detection Tools (ART) implemented in CONN. We enforced conservative motion censoring thresholds, scrubbing frames exceeding > 0.5 mm frame-wise motion or Global Signal *z* > 3. At this stage, 14 of the 135 participants were dropped from the 7T analyses for having fewer valid scans than the rest of the group (as defined by 1st Q − 1.5 IQR; see Supplementary Figure 1). Thus, the final sample to be considered in 2nd-level analysis consisted of 121 participants.

The functional data were further denoised using the CONN Toolbox’s *aCompCor* method (white matter and CSF noise, frame-wise motion regression, artifact scrubbing, and linear detrending). The data were then band-pass filtered to isolate resting-state frequencies (0.01 Hz < *f* < 0.10 Hz) using a fast Fourier transform (FFT). Importantly, the 7T seed region of interest BOLD timeseries were extracted from resulting unsmoothed and denoised rsfMRI volumes to prevent “spillover” from other nearby regions. For the 3T dataset, masks were applied to smoothed data, which have higher signal-to-noise ratio. Denoising was successful in this final sample, eliminating the positive skew and inter-subject variability related to physiological and motion artifact in randomly sampled functional connectivity (Supplementary Figure 2) and there was no evidence for a link between mean motion or mean global signal change and functional connectivity in the 7T or 3T datasets (see Supplementary Figures 3, 4).

#### Seed Regions

**Figure 1:**
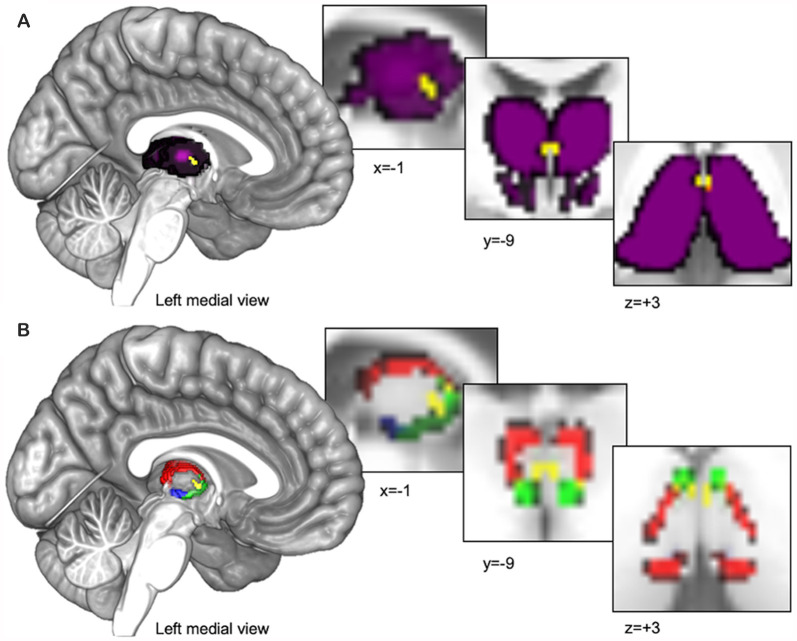
Thalamus seed regions entered into functional connectivity analyses. **(A)** The Paraventricular Thalamic Nucleus (PVT) seed (shown in yellow) surrounded by the rest of the thalamus (shown in purple). The purple seed region encompassing the rest of the thalamus was used for the analyses presented in Figures 2–Figures 4. **(B)** In separate analyses, we also compared PVT connectivity with nearby midline thalamic subnuclei CeM (green), CL (red), and the Pf (blue; see Figures 5, 6).

We utilized 3D digital seed masks from the “Thalamus Atlas” of the Swiss Federal Institute of Technology (ETH) in Zurich and University of Zurich, Switzerland (Krauth et al., 2010; Jakab et al., 2012). The digital model was utilized with written consent from the Computer Vision Laboratory of the ETH Zurich. The atlas consists of seed masks of human thalamic subdivisions defined using histology (Krauth et al., 2010) and implemented in standard Montreal Neurological Institute (MNI) voxel space for functional neuroimaging (Jakab et al., 2012). We combined the left and right hemisphere masks into single bilateral masks for each subnucleus to increase coverage and total voxel count. We used the bilateral PVT mask (Pv.nii.gz, 14 mm^3^ shown in yellow in Figures 1A,B) as our primary seed of interest and we used the remainder of the thalamus in our primary semi-partial control analyses (see purple region in Figure 1A) as well as other nearby medial group subnuclei [central medial nucleus (CeM.nii.gz, 327 mm^3^ see green region in Figure 1B), central lateral nucleus (CL.nii.gz, 1740 mm^3^, see red region in Figure 1B), and the parafascicular nucleus (Pf.nii.gz, 380 mm^3^, see blue region in Figure 1B), separately] in a separate set of analyses focused on controlling for and comparing the PVT functional connectivity to that of other nearby midline thalamic subnuclei with some similarities and differences in their projection profiles in rodents (Van der Werf et al., 2002).

To avoid the loss of small ROI information in the 1.6 mm and 2 mm functional spaces, the CONN Toolbox whole-brain seed-to-voxel analysis extracts and averages the BOLD timeseries from the closest corresponding voxels (e.g., 14 total voxels for the PVT) in the functional maps to correlate with the non-PVT voxels in the smoothed maps. This approach, as opposed to first resampling the small ROI subnuclei, guarantees an appropriate partial-volume weighting of the functional data and no loss of smaller ROIs like the PVT or the subnuclei.

The digitized subnuclei regions (e.g., PVT, CeM, CL, Pf) were received in standard MNI space. SPM12’s *imcalc* function was used to combine the left and right seeds into 1 mm bilateral masks and no other transformations were performed. The whole-thalamus mask (global.nii.gz) provided by Krauth et al. (2010) and MNI-aligned by Jakab et al. (2012) covers the entire thalamus and naturally includes the PVT. To make the remainder of the thalamus or rest-of-thalamus seed (minus the PVT), we first added the 0.5 mm left and right global.nii.gz files to make a bilateral global thalamus seed. To have more control over the larger rest-of-the-thalamus ROI in the functional space and view the subtraction of the PVT from the ROI, we resampled the Krauth et al. (2010) masks (Pv.nii.gz and global.nii.gz) masks to the 7T functional space (1.6 mm) and slightly dilated the PVT region once using MRICRON^3^. Finally, the dilated PVT region was subtracted from the global thalamus region to make the purple region shown in Figure 1A. The exact same seed regions were used in the 7T and 3T analyses.

#### Subject-Level Bivariate and Multivariate Seed-Based Connectivity

The goal of the current study was to identify resting state functional connectivity of the PVT, with a focus on connectivity patterns that withstand controlling for signals from the rest of the thalamus or other nearby midline subnuclei. First, whole-brain standard bivariate correlations of the PVT alone were assessed separately in a seed-based functional connectivity (SBC) analysis. Next, multivariate seed-based connectivity (mSBC) was calculated as the semipartial correlation coefficients between the BOLD timeseries of the PVT and all other individual voxel timeseries in the brain after controlling for the BOLD timeseries of the other seeds (e.g., the rest of the thalamus in a first analysis and specific midline thalamic subnuclei in a separate second analysis; Whitfield-Gabrieli and Nieto-Castanon, 2012; Nieto-Castanon, 2020). That is, unique contributions of functional connectivity were calculated by entering the seed masks jointly into a general linear model. Both analyses produced Fisher *r*-to-*Z* transformed whole-brain maps of PVT functional connectivity representing the transformed correlation and semipartial correlation coefficients for each seed region and subject. Subject-level functional connectivity SBC and mSBC maps were calculated separately for both rsfMRI sessions and were then averaged across sessions. These subject-level connectivity maps were then entered into separate group-level analysis of the SBC and mSBC maps. All rsfMRI analyses were conducted in standardized MNI space.

#### Group-Level Analysis and Thresholding

##### Threshold-Free Cluster Enhancement (TFCE)

Group functional connectivity statistics were calculated as voxel-level cluster-inferences using TFCE (Smith and Nichols, 2009) implemented in the CONN Toolbox. TFCE uses voxel-wise cluster-wise evidence and offers greater sensitivity than other thresholding methods without the need to define an arbitrary initial cluster-forming threshold or voxel extent. Voxel-wise TFCE scores reflect both the strength of the statistical effect at each voxel and cluster-like local spatial support (Smith and Nichols, 2009). Results were considered significant at* p*TFCE-FWE < 0.05 (estimated using 1,000 permutation iterations of the data).

##### PVT Connectivity Compared to the Rest of the Thalamus

*Semi-Partial Correlations*. To demarcate regions correlated with the PVT (bivariate correlations) that also survived controlling for average signal from the rest of the thalamus (semi-partial correlations), we created the conjunction of the thresholded SBC and mSBC maps using *imcalc* implemented in SPM12 [i.e., PVT functional connectivity (bivariate map) ∩ PVT functional connectivity controlling for the rest of the thalamus (semi-partial map)]. In order to report the 7T positive and negative FC results we calculated voxel count and percent overlap within each labelled region of The Human Brainnetome Atlas (Fan et al., 2016), which contains many of the regions of interest except for the VTA, BNST, PAG, or hypothalamus (HYPO). As such, we also assessed overlap between FC maps and regions of interest including 2 mm spheres created around coordinates corresponding to the lateral and medial hypothalamic areas (see MNI coordinates in Table 5 of Baroncini et al., 2012), the LC (Keren et al., 2009; LC_2SD_BINARY_TEMPLATE.nii download from http://eckertlab.org/LC/) as well as probabilistic maps of the VTA (thresholded at 80% probability; Murty et al., 2014), BNST (thresholded at 50% probability; from Torrisi et al., 2015), and PAG (PAG_prob_mni_linear_young.nii from Keuken et al., 2014).

To quantify similarity of PVT functional connectivity with known large-scale cortical resting-state networks (Thomas Yeo et al., 2011), we calculated cosine similarity using publicly available code from Cornblath and colleagues (Cornblath et al., 2020, see https://github.com/ejcorn/brain_states) between the positive functional connectivity and negative functional connectivity components of the resulting network of semi-partial statistics of the PVT (controlling for the rest of the thalamus) and the PVT (controlling for the Pf, CeM, and CL). The three subnuclei control seeds were also assessed for their macrostructure functional connectivity. First, for each of the 210 cortical regions of The Human Brainnetome Atlas, we extracted the *T* value of FC from the unthresholded statistical map of the semi-partial correlation results, controlling for the average functional connectivity of the rest of the thalamus. This yielded a 210 × 1 vector quantifying unique PVT FC in each cortical region (see Figure 4A). This vector of functional connectivity values was entered into a cosine similarity analysis comparing the functional connectivity values to seven separate 210 × 1 binary vectors representing membership to a known large-scale networks (network membership of BN atlas regions can be found in subregion_func_network_Yeo.csv at https://atlas.brainnetome.org/download.html). The network binary vectors are depicted in Figure 4B. Cosine similarity was then calculated for each network and displayed using polar plots. The highest cosine similarity values for positive FC and negative FC, separately, were used to determine the best network match.

**Figure 2:**
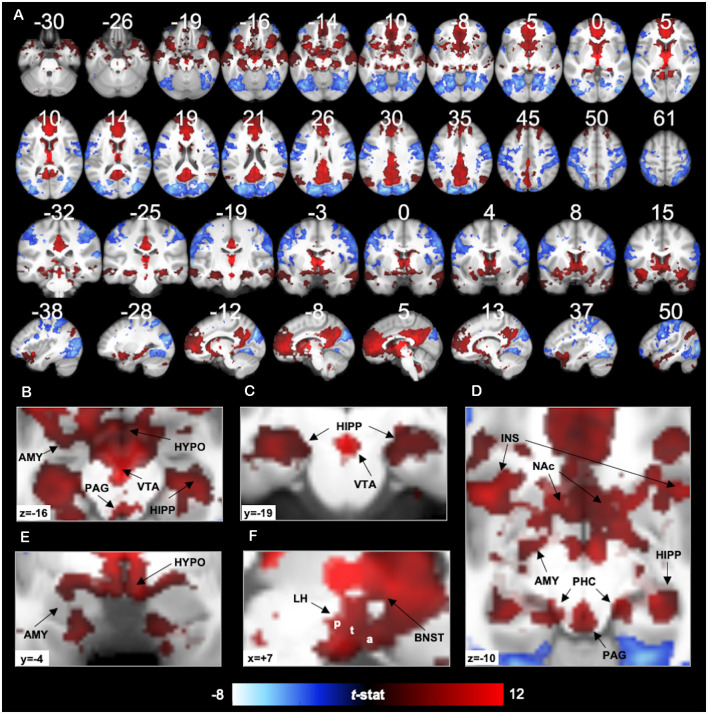
7T PVT functional connectivity. Positive (red) and negative (blue) functional connectivity of the PVT controlling for average signal from the rest of the thalamus. Results thresholded at *p*TFCE-FWE < 0.05. Statistical overlays shown on average subject anatomical. **(A)** Multi-planar views (compare to the 3T results in Figure 7 and Supplementary Figures 9, 10 shown on the same slices). **(B)** Axial slice highlighting positive FC with the amygdala (AMY), hippocampus (HIPP), Hypothalamus (HYPO), Periaqueductal Gray Area (PAG), and Ventral Tegmental Area (VTA). **(C)** Coronal slice showing positive FC with the bilateral hippocampus and the VTA. **(D)** Axial slice showing positive FC with AMY, HIPP, insula (INS), nucleus accumbens (NAc), PAG, and parahippocampal cortex (PHC). **(E)** Coronal slice showing positive FC with the AMY and HYPO. **(F)** Sagittal slice showing lateral HYPO (a = anterior, t = tuberal, p = posterior) and Bed Nucleus of the Stria Terminalis (BNST) positive FC. See Supplementary Figure 6 for further illustrations of positive FC with BNST, HYPO, PAG, and VTA.

**Figure 3:**
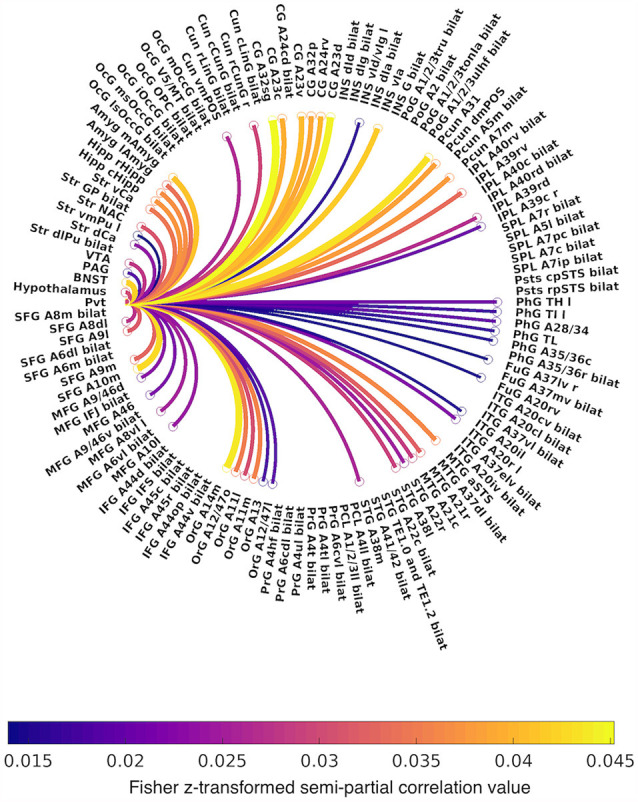
Circular graph summarizing positive functional connectivity of the PVT, controlling for the rest of the thalamus. Lines are color-coded by Fisher *z*-transformed semi-partial correlation values. Abbreviations: A, area; CG, cingulate gyrus; Cun, cuneus; d, dorsal; IFG, inferior frontal gyrus; INS, insula; IPL, inferior parietal lobule; ITG, inferior temporal gyrus; FuG, fusiform gyrus; l, lateral; m, medial; MFG, middle frontal gyrus; MTG, middle temporal gyrus; OcG, occipital gyrus; OrG, orbital gyrus; PrG, precentral gyrus; PCL, paracentral lobule; Pcun, precuneus; PhG, parahippocampal gyrus; PoG, postcentral gyrus; SFG, superior frontal gyrus; SPL, superior parietal lobule; STG, superior temporal gyrus; Str, striatum; v, ventral.

**Figure 4:**
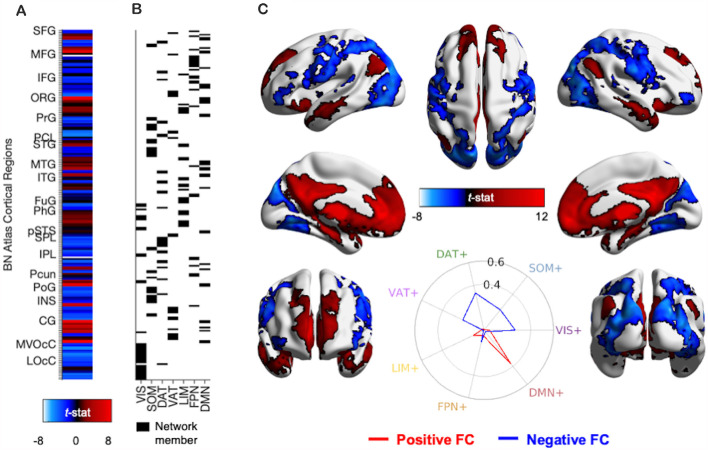
Methods and results for identifying PVT functional connectivity macro-structures. **(A)** Semi-partial correlation statistics of PVT FC, controlling for the rest of the thalamus, were extracted from the 210 cortical regions of the BN Atlas to create an FC vector. **(B)** Cosine similarity between the positive FC values (shown in red) and negative FC values (shown in blue) and each of the seven binary vectors representing group membership to a 7-parcellation scheme from Thomas Yeo et al. (2011) were calculated separately. **(C)** Thresholded network rendered view of the same PVT functional connectivity maps shown in Figure 2. Positive (red) and negative (blue) functional connectivity of the PVT, controlling for average signal from the rest of the thalamus. Results thresholded at *p*TFCE-FWE < 0.05. Inset polar plot depicts the results of the cosine similarity between the positive functional connectivity (red) and negative functional connectivity (blue) map statistics with binary vectors corresponding to known rest-state networks (Thomas Yeo et al., 2011). The positive FC correspondence is observable between the red region rows in **(A)** and the Default Mode Network (DMN) network membership column in **(B)**. Polar plots created using code from Cornblath et al. (2020). Abbreviations: CG, cingulate gyrus; DAT, dorsal attention network; DMN, default mode network; FPN, fronto-parietal network; FuG, fusiform gyrus; IFG, inferior frontal gyrus; INS, insula; IPL, inferior parietal lobule; ITG, inferior temporal gyrus; LIM, limbic network; LOcC, lateral occipital cortex; MFG, middle frontal gyrus; MTG, middle temporal gyrus; MVOcC, medio-ventral occipital cortex; OrG, orbital gyrus; PCL, paracentral lobule; Pcun, precuneus; PhG, parahippocampal gyrus; PoG, post-central gyrus; PrG, pre-central gyrus; pSTS, posterior superior temporal sulcus; SFG, superior frontal gyrus; SOM, somato-motor network; SPL, superior parietal lobule; STG, superior temporal gyrus; VAT, ventral attention network; VIS, visual network.

##### PVT Connectivity Compared to Other Midline Thalamic Subnuclei

Controlling for the average signal from the remainder of the thalamus (i.e., thalamus without the PVT mask) using semi-partial correlations demarcated unique functional connectivity while controlling for average thalamic signal. However, to reveal functional connectivity that was stronger for the PVT compared to other midline areas we conducted further analyses comparing semi-partial PVT correlations individually to other nearby medial subnuclei (CeM, CL, and Pf, see Figure 1B). For instance, we tested the conjunction of the thresholded SBC maps using SPM12 *imcalc* (e.g., PVT bivariate map ∩ PVT > CL semi-partial map both thresholded separately at *p*TFCE-FWE < 0.05). We conjoined the maps to ensure activity differences were not driven by a strong negative correlation with the comparison region. We also conducted a supplementary whole-brain pairwise comparison of bivariate connectivity that was significantly greater for the PVT compared to the rest of the thalamus [i.e., PVT functional connectivity (bivariate) ∩ PVT > Rest of the thalamus]. It is important to note that there are likely substantially different temporal signal-to-noise ratios for the signal measured from 14 cubic mm PVT voxels compared to the other subnuclei.

#### Data Visualization

The TFCE maps used to identify significant voxels were binarized and then applied to the SBC unthresholded *t*-stat maps for visualization purposes (i.e., colorbars in figures represent *t*-values instead of TFCE values). Visualization of the statistical maps were created using MRIcroGL^4^ and with the BrainNet Viewer^5^ while using the package to make publicized pictures (Xia et al., 2013). The polar plot illustrating cosine similarity between PVT connectivity and similarity to known resting state networks were created using publicly available code from Cornblath and colleagues^6^. Raincloud plots were created using code from Allen and colleagues (Allen et al., 2019).

## Results

### 7T Dataset

#### PVT Functional Connectivity With Specific Structures

The first analysis examined PVT functional connectivity that survived partial correlation with functional connectivity from the rest of the thalamus (i.e., the conjunction of the bivariate and semi-partial maps of PVT connectivity). The results of the 7T group analysis are shown in Figures 2–4. The results are summarized in Table 1 and in a circular graph that depicts the positive FC statistic for each of the BN Atlas regions that reach significance and the smaller brainstem and midbrain regions (Figure 3). The full details of the percent coverage of each region of within BN Atlas are reported in Supplementary Table 2 (positive FC) and Supplementary Table 3 (negative FC).

**Table 1:** Summary table of brain regions that show significant positive FC with the PVT, controlling for the rest of the thalamus (corresponds to positive FC results shown in Figures 2–4).

Lobe	Gyrus	Modified cyto-architectonic
Frontal	Middle frontal gyrus (MFG)	Area 46, dorsal area 9/46, lateral area 10, ventrolateral area 8.
	Orbital gyrus (OrG)	Area 13, orbital and lateral area 12/47 and medial 14, medial and lateral area 11 (including vmPFC).
	Superior frontal gyrus (SFG)	Dorsolateral area 8, medial area 10, medial and lateral area 9 (including dmPFC).
	Insular (INS)	Ventral agranular and granular insula.
Limbic	Cingulate gyrus (CG)	Subgenual area 32, pregenual area 32, caudal and dorsal area 23, rostroventral area 24, ventral area 23.
Occipital	Cuneus (Cun)	Rostral cuneus gyrus, ventromedial parietooccipital sulcus.
Parietal	Inferior parietal lobule (IPL), Angular gyrus	Caudal area 39, rostrodorsal area 39, rostroventral area 39.
	Precuneus (Pcun)	Area 31, dorsomedial parietooccipital sulcus, medial area 7.
Subcortical	Amygdala (Amyg)	Medial and lateral amygdala.
	Hippocampus (Hipp)	Caudal and rostral hippocampus.
	Striatum (Str)	Dorsal and ventral caudate, nucleus accumbens, ventromedial putamen.
Temporal	Fusiform gyrus (FuG)	Rostroventral area 20, lateroventral area 37.
	Inferior temporal gyrus (ITG)	Intermediate lateral area 20, rostral area 20.
	Middle temporal gyrus (MTG)	Anterior superior temporal sulcus, caudal area 21, rostral area 21.
	Parahippocampal gyrus (PhG)	Area 28/34 (EC, entorhinal cortex), area TI (temporal agranular insular cortex), area TL (lateral PPHC, posterior parahippocampal gyrus), area TH (medial PPHC), caudal area 35/36.
	Superior temporal gyrus (STG)	Medial and lateral areas 22 and 38 (temporal pole).
Other	Brainstem and midbrain	Bed nucleus of the stria terminalis (BNST), hypothalamus, periaqueductal gray (PAG), ventral tegmental area (VTA).

*For more information on cyto-architectonic labeling see the guide to The Human Brainnetome Atlas (Fan et al., 2016). For more details of voxel extent and percent coverage of each BN Atlas region see Supplementary Tables 2, 3. Area label numbers are similar to that of Brodmann areas*.

TFCE analysis of only the SBC bivariate map of the PVT identified three large clusters with significant positive functional connectivity and two clusters with significant negative functional connectivity (see Supplementary Table 1 and Supplementary Figure 5 for cluster statistics and information).

As expected, positive functional connectivity was observed with portions of the brainstem, hypothalamus, and basal forebrain (see Figures 2B–F). The positive functional connectivity map overlapped with the multiple subnuclei of the hypothalamus, including MNI coordinates corresponding to the lateral hypothalamic area (including the anterior, tuberal, and posterior areas of the lateral hypothalamus), ventro- and dorso-medial hypothalamic nuclei, supraoptic nucleus, mammillary nucleus, and posterior hypothalamus (Baroncini et al., 2012). In the VTA, there was 38% overlap between the positive functional connectivity map and the VTA region defined by Murty et al. (2014). We also observed PVT positive functional connectivity in 94% of voxels in the probabilistic mask of the BNST (e.g., coverage near MNI_xyz_ = ±5, 4, −2) and 88% of PAG voxels bilaterally. Supplementary Figure 6 depicts the exact voxels of overlap with probabilistic atlas maps of these smaller regions. There was very minimal overlap with the LC mask.

In the striatum, unique positive functional connectivity of the PVT was observed in the NAc (see Figure 2D) and the dorsal and ventral portions of the caudate (see Figure 2A sagittal slices). In the MTL, positive functional connectivity was observed extensively along the anterior-posterior extent of the hippocampus—with more extensive coverage of the anterior hippocampus—and with the amygdala bilaterally (see Figures 2A–E). There were also suprathreshold voxels observed in the parahippocampal cortex (PHC; see Figure 2D), including more than half of the entorhinal cortex. As expected, we observed positive functional connectivity with the insula (see Figures 2A,D), particularly in voxels corresponding to the ventral agranular area.

Cortically, positive functional connectivity was observed in large swaths of the medial PFC, precuneus, and cingulate as well as the angular gyrus, middle and superior temporal gyrus, and the temporal pole. The coverage in the cingulate included the sub-genual cingulate area.

Negative functional connectivity was observed with portions of visual processing cortex (including the cuneus, lingual gyrus, superior and middle occipital gyri, lateral occipital cortex, fusiform gyrus, inferior temporal gyrus), superior parietal lobule, inferior parietal lobule (including the supramarginal and angular gyri), somato-motor areas (including the pre- and post-central gyrus), and ventro-lateral PFC/inferior frontal gyrus. Negative fluctuations were also observed with the insula, mostly in the dorsal dysgranular area. See activity displayed in blue in Figures 2, 4 and Supplementary Table 3.

#### PVT Functional Connectivity With Macro-structures

**Figure 5:**
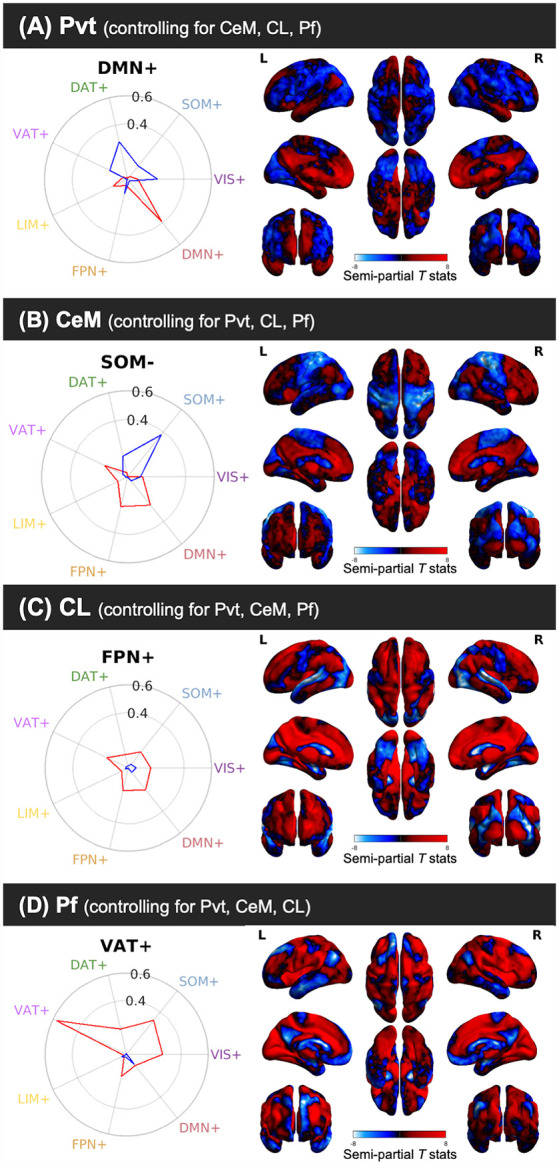
Cosine similarity between binary vectors representing a 7-Network Parcellation of large-scale functional networks (Thomas Yeo et al., 2011) and the positive FC (red) and negative FC (blue) patterns for the semi-partial correlation statistics for the PVT, CeM, CL, and Pf. Polar plots are shown on the right and the unthresholded semi-partial correlation maps are shown on the right. **(A)** The PVT showed the highest cosine similarity with the DMN+/DAT-. **(B)** The CeM positive FC also showed correspondence with the DMN+, although numerically weaker than the PVT. The CeM negative FC best matched with SOM-. **(C)** The CL positive FC map did not show a strong cosine similarity with any of the known large-scale intrinsic networks, but was numerically best-matched with the FPN+. **(D)** The Pf showed the strongest cosine similarity with the VAT+. Abbreviations: DAT, dorsal attention network; DMN, default mode network; FPN, fronto-parietal network; LIM, limbic network; SOM, somato-motor network; VAT, ventral attention network; VIS, visual network.

We used cosine similarity analysis to determine which known intrinsic functional connectivity network (Thomas Yeo et al., 2011) or macro-structure best captured the observed PVT functional connectivity pattern. The positive functional connectivity pattern of the PVT was most strongly matched with the DMN+ (see red line, inset polar plot of Figure 4). The negative FC pattern was dispersed across several networks, but was most closely matched with the Dorsal Attention Network (DAN) (see blue line on polar plot in Figure 4), a network known to be anti-correlated with the DMN+. A similar pattern for the PVT was observed when controlling for the three midline thalamic subnuclei control regions (see Figure 5A). To ensure DMN+ network membership was not generic to the thalamus, we repeated the cosine similarity analysis for each of the three control subnuclei. Specifically, we permutated through each of the different control subnuclei semi-partial FC maps to characterize their FC patterns with functional networks, while controlling for the other three regions (e.g., CL controlling for CeM, Pf, and PVT). The positive FC patterns of the Pf, CL, and CeM were best matched with the ventral attention network (VAN+), fronto-parietal network (FPN+), and FPN/DMN+ networks, respectively (see Figures 5B–D). However, the PVT FC pattern was a numerically better match with the DMN+ than that of the CeM [cosine similarity value: 0.4 (PVT) vs. 0.25 (CeM)]. These data suggest the PVT is functionally linked with the DMN+ at rest and this pattern is not generic to midline thalamic subnuclei.

#### Direct Comparison of PVT Connectivity to Control Seeds

**Figure 6:**
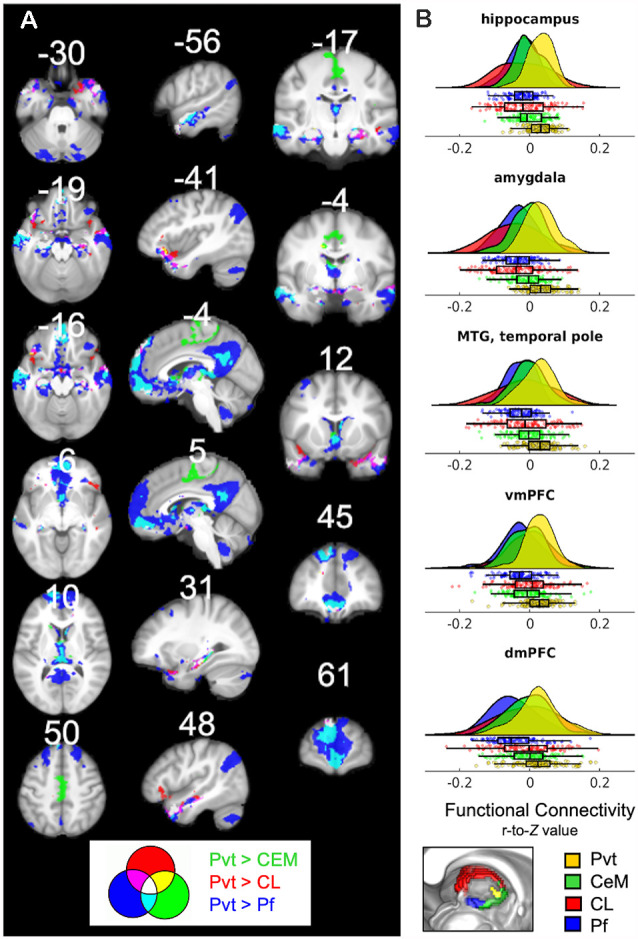
**(A)** Regions that show positive PVT functional connectivity that is greater than functional connectivity of the CeM (green), CL (red), or Pf subnuclei (blue; e.g., PVT bivariate map ∩ PVT > CL semi-partial map both thresholded separately at *p*TFCE-FWE < 0.05). Some areas showed greater functional connectivity compared to CL and CeM (yellow), CeM and Pf (cyan), CL and Pf (violet), or all three (white). **(B)** Raincloud plots display density plots with the individual Fisher *r*-to-*Z* connectivity values for each participant extracted from regions that showed overlap between all three maps. Functional connectivity estimates from the separate ROIs are displayed in colors corresponding to the legend at the bottom. From top-to-bottom the subplots are displayed in descending order based on average effect size as calculated by paired Cohen’s d. Inset color mixing image (lower left) accessed from https://colourware.wordpress.com/ (colourmixing.gif, 2013).

Thus far, the results report on significant PVT functional connectivity that survives controlling for functional connectivity of the control regions and represents unique functional connectivity. We next tested where PVT functional connectivity exceeded that of the control regions. Whole-brain comparisons of the semi-partial correlations for the PVT compared to the CeM, CL, and Pf, separately, showed greater positive PVT functional connectivity with the amygdala, hippocampus, ventromedial PFC/orbital frontal gyrus (Brodmann area 11) and dorsomedial PFC (Brodmann area 9 and 10), left lateral orbital frontal gyrus (Brodmann area 47), and middle temporal gyrus/temporal pole [see overlap of the three maps shown in white (left) and corresponding raincloud plots (right) in Figure 6]. Voxels in the NAc were found in the maps of PVT > Pf and to a lesser extent PVT > CeM, but not Pf > CL.

A similar pattern was observed for the whole-brain comparison of PVT > Rest of the Thalamus (Supplementary Figure 7). However, given the size differential between the PVT seed and the rest of the thalamus seed, we focused on the whole-brain comparisons to the relatively smaller subnuclei seeds. Further, control analyses returned similar converging results in the hippocampus, amygdala, and middle temporal gyrus when smaller sub-portions of the subnuclei control regions were seeded to be approximate the size of the PVT (e.g., a smaller 20 mm^3^ CL seed; see Supplementary Figure 8). Together, these results suggest that PVT resting FC with the hippocampus and amygdala is strong relative to nearby midline regions (but see “Limitations and Future Directions” section).

### 3T Dataset

**Figure 7:**
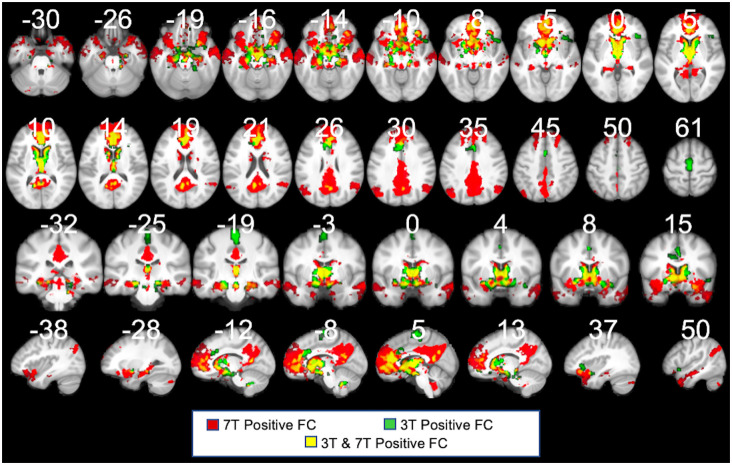
3T PVT positive functional connectivity (green) showed with the 7T results (shown in red). Regions shown in yellow demarcate regions that showed positive FC in both the 7T and 3T datasets. Each maps controls for the average signal from the rest of the thalamus. All results thresholded at *p*TFCE-FWE <0.05.

Recognizing that the 3T FC maps are likely noisier than the 7T maps, we nonetheless sought to test the PVT functional connectivity in the 3T data of the same participants. Indeed, the positive FC 3T maps (shown in green in Figure 7) were noisier and less sensitive than the 7T maps (shown in red). However, there were substantial consistencies between the 7T and 3T positive FC maps (shown in yellow in Figure 7), particularly in the mPFC, precuneus, striatum (NAc and caudate), MTL areas (amygdala and hippocampus), anterior insula, VTA, and hypothalamus, but less coverage along the frontal pole/dmPFC and in the angular gyrus, middle temporal gyrus, and temporal pole, and lateral areas. The 3T positive and negative FC maps are shown in Supplementary Figure 9 and their overlap with the 7T results in Supplementary Figure 10. These results highlight the limitations of the PVT positive FC at 3T relative to 7T, but also emphasize the robustness of the major connectivity patterns across different magnet strengths.

All thresholded and unthresholded 3T and 7T maps can be viewed in Supplementary Figure 11.

## Discussion

The present study utilized high-quality, long-duration, high-resolution 7T rsfMRI data from The Human Connectome Project in conjunction with thalamic subnuclei masks derived from multiple-histological data to demarcate resting state functional connectivity patterns of the human PVT. We observed pronounced PVT functional connectivity with the brainstem, midbrain, ventral striatum, MTL (including the hippocampus and amygdala), cingulate, mPFC, and lateral temporal cortex. The principal findings of the current study are that resting human PVT functional connectivity shows: (1) substantial overlap with the known anatomical and functional connectivity shown in animal PVT, (2) distinctive connectivity patterns with subcortical and cortical structures compared to the average thalamus signal and other nearby midline-thalamic regions, and (3) appears to be linked at rest with the DMN. These findings are largely consistent with the known structural and functional connectivity of the PVT in experimental animals, highlights important cortical nodes in humans, and additionally situates the PVT within the resting DMN. Here we discuss the results of PVT functional connectivity with specific brain regions and networks as well as potential relevance to psychopathology.

### PVT Connectivity With Specific Structures

#### Brainstem and Midbrain

The PVT showed positive resting functional connectivity with the hypothalamus at coordinates corresponding to the lateral and medial hypothalamus (Baroncini et al., 2012), consistent with prior work (Moga et al., 1995; Van der Werf et al., 2002; Vertes and Hoover, 2008). The lateral hypothalamus projects to the PVT and has been associated with anxiety, stress responses—including adaption of the stress response following chronic stress (Bhatnagar et al., 2002)—and wakefulness *via* projections to NAc (Ren et al., 2018; Barson et al., 2020). We also observed positive functional connectivity of the PVT with the BNST—a region that receives projections from the PVT (Bhatnagar and Dallman, 1998; Millan et al., 2017) associated with anxiety (Li and Kirouac, 2008; Vertes and Hoover, 2008; Dong et al., 2017). There was also evidence of PVT functional connectivity with the PAG, a known connection in rodents (Krout and Loewy, 2000) functionally thought to support integration of visceral information (Li and Kirouac, 2008). The PVT-VTA functional connectivity is also consistent with tracing studies (Vertes et al., 2015). Prior work has shown that pharmacological activation of the PVT *in vivo* induces dopaminergic neuron activity in the VTA, and that converging inputs of the hippocampus and PVT on the NAc regulate dopamine in the VTA (Li and Kirouac, 2008). However, recent work suggests dopaminergic fibers in the PVT originate from the hypothalamus and PAG or stress-induced dopamine modulation from the LC (Li et al., 2014; Beas et al., 2018; Fraser and Janak, 2018), and not from traditional dopaminergic centers such as the VTA. Although the current work does not measure reward-related activation of this circuit, the results demonstrate stationary correlations between these regions and the PVT at rest, consistent with the underlying circuitry. Future work using neuromelanin MRI procedures and tracing protocols (e.g., Clewett et al., 2016) to image the LC is needed to further test the resting and stress-related responsivity in this node of the circuit (Berendse and Groenewegen, 1991; Witter, 2006; Li and Kirouac, 2008).

#### Striatum

As expected, the PVT was functionally connected with the NAc. PVT-NAc connections are thought to be associated with reward and motivation (Barson et al., 2020). A PVT stimulation releases dopamine in the NAc (Parsons et al., 2007) and self-stimulation of these connections are thought to be rewarding (Li and Kirouac, 2008; Vertes and Hoover, 2008; Dong et al., 2017). In rodents, NAc inputs from the PVT are classically known to be glutamatergic (Li and Kirouac, 2008; Vertes and Hoover, 2008; Dong et al., 2017) but recent work demonstrates corticotropin releasing hormone (CRH)-expression NAc afferents in the PVT (Itoga et al., 2019). Specifically, thalamic nuclei comprise the origin of a third of the CRH projections to the NAc, and PVT accounts for a third of all thalamic NAc-projecting cells (9.40% of the total). These data predict that even stronger NAc-PVT functional connectivity could be revealed during suitable tasks. Consistent with prior work in rodents, we also observed functional connectivity of the PVT with the caudate (Van der Werf et al., 2002; Vertes and Hoover, 2008), a region shown to have relatively stronger connectivity with the posterior PVT in rodents (Li and Kirouac, 2008).

#### Medial Temporal Lobe

The functional connectivity of the PVT with the hippocampus and amygdala was consistently implicated in all analyses, including the whole-brain comparisons of FC strength compared to that of the nearby subnuclei control regions. These findings are aligned with prior work showing dense connectivity between the PVT and the hippocampus and amygdala and contribute to accumulating evidence that the PVT is functionally well-positioned to subserve emotional memory functions (Su and Bentivoglio, 1990; Do-Monte et al., 2016). The demarcation of the hippocampus along the extent of its long axis was particularly notable, with ~80% coverage of the anterior hippocampus and ~50% of the posterior hippocampus. The dorsal and ventral hippocampi in rodents—which correspond to the posterior and anterior hippocampus in primates—are associated with cognitive and emotional/stress functions, respectively (Fanselow and Dong, 2010). Prior work has shown strong reciprocal connectivity between the ventral subiculum of the hippocampus and the PVT (Berendse and Groenewegen, 1991; Witter, 2006; Li and Kirouac, 2008), particularly with the aPVT (Su and Bentivoglio, 1990; Witter, 2006; Li and Kirouac, 2012), as well as connectivity with the entorhinal and parahippocampal cortices (Van der Werf et al., 2002), also observed here in PVT functional connectivity.

The amygdala, particularly the central amygdala, is a major PVT target (Li and Kirouac, 2008; Vertes and Hoover, 2008; Dong et al., 2017). Projections largely from the pPVT to the central amygdala form a connection functionally associated with the consolidation, maintenance, and expression of long-term fear memories (Do-Monte et al., 2015, 2016; Penzo et al., 2015; Chen and Bi, 2019) as well as the sleep-wake cycle, reward and motivation, and anxiety (Barson et al., 2020). While the basolateral amygdala is required for early fear memory retrieval, the PVT becomes increasing important for fear memory after 24 h (Do-Monte et al., 2016). These studies suggest a time-dependent role of the PVT in retrieving long-term, well-consolidated fear memories—rather than retrieval of younger memories—and less of a role during the learning or encoding phase (Padilla-Coreano et al., 2012; Do-Monte et al., 2016). Unlike the basolateral amygdala, which plays a more general Pavlovian role in fear and motivated learning, the recruitment of the PVT might serve to coordinate salient memories with biologically adaptative responses and homeostatic regulation (Do-Monte et al., 2016). Together, the findings demonstrate that the PVT is functionally coupled with the MTL and ventral striatum, which might enable the ability to control and engage appetitive or aversive motivational behaviors under conflict when a conditioned stimulus presents mixed motivational valence (Choi et al., 2019).

#### Cerebral Cortex

The positive functional connectivity maps also covered large swaths of medial PFC, including vmPFC/medial orbital cortex, dmPFC, subgenual area 25 and dorsal areas of the anterior cingulate, In rodents and non-human primates, the PVT has strong reciprocal connections with the IL and PL (medial PFC; Chiba et al., 2001; Vertes, 2004; Price and Drevets, 2012), with stronger connectivity between the IL and PL with the anterior and posterior PVT, respectively (Barson et al., 2020; Gao et al., 2020). Based on their structural connectivity patterns, the IL and PL areas in rodents are thought to be similar to areas of mPFC in humans. Portions of these mPFC areas showed greater positive functional connectivity of the PVT compared to the three control thalamic subnuclei. Recent work has demonstrated that the PVT gates arousal-related activation of the vmPFC and that activity in the PVT-vmPFC loop is inversely related to arousal (Gao et al., 2020). Our findings of resting PVT-vmPFC functional connectivity is consistent with greater activity between these areas during periods of low arousal (e.g., a resting state). However, under challenge to the system during a task, excitation of the mPFC by the PVT by glutamatergic projections might increase cortical arousal and direct attention to visceral or emotional states (Huang et al., 2006).

Consistent with prior work, we observed PVT functional connectivity with the anterior agranular insula (Berendse and Groenewegen, 1991), which receives relatively stronger innervation from the pPVT in rodents (Vertes and Hoover, 2008; Vertes et al., 2015). The anterior insula is known for its role in integrating viscero-sensory and gustatory signals. In humans, the ventral anterior portion of the insula, as observed here, has been functionally associated with emotional processes (e.g., fear, happiness, anger, sadness, and disgust; Kelly et al., 2012), awareness of bodily feelings (Craig, 2011), autonomic responses and visceral states (Mutschler et al., 2009; Critchley and Harrison, 2013), as well as attentional, socioemotional, salience processing, and gustatory processing (Uddin et al., 2017). Functional connectivity with multisensory insula might facilitate and integrate signals related to salience, emotional states, and cue-reward associations with ongoing autonomic arousal to influence motivated behaviors (Kirouac, 2015).

### PVT Connectivity With the Default Mode Network

In addition to positive functional coupling with discrete brain regions, we investigated the possibility that the PVT is functionally linked with distinct, intrinsic macrostructures, such as the DMN, a network strongly associated with self-referential thought/affective decisions as well as episodic memory retrieval, and the subjective experience of memory (Andrews-Hanna et al., 2010; Kim, 2020). The DMN is comprised of a midline “core” (mPFC and posterior cingulate) and two subsystems: the Dorsomedial Prefrontal Cortex Subsystem (DMPFC: temporal pole, lateral temporal cortex, temporal-parietal junction, and dmPFC) and the Medial Temporal Lobe Subsystem (hippocampus, parahippocampal cortex, retrosplenial cortex, posterior inferior parietal lobule, and the vmPFC), involved in self-referential processing/affective decisions and memory, respectively (Andrews-Hanna et al., 2010).

Here, we used the cortical network parcellations by Thomas Yeo and colleagues (Thomas Yeo et al., 2011, see Figure 7) to quantify the best match between the cortical areas of the PVT positive FC map and the 7-network parcellation. In the positive functional connectivity maps, we observed PVT functional connectivity with areas of the DMN “Core” (mPFC and posterior cingulate) as well as the DMPFC Subsystem (including the dmPFC, lateral temporal cortex/middle temporal gyrus, temporal pole) and the MTL Subsystem (includes the vmPFC, parahippocampal cortex, hippocampus, and angular gyrus of the inferior parietal lobule). Indeed, while controlling for functional connectivity patterns of the rest of the thalamus (see Figure 3) as well as the other thalamic subnuclei (Figure 4), the PVT showed the strongest match with the DMN+ network as parcellated by Thomas Yeo et al. (2011). These analyses demonstrated that PVT was not simply functionally connected to the DMN+ because the participants were in a resting state, because other subnuclei were more closely matched with other more task-positive networks. Similarly, recent work showed resting habenular nucleus functional connectivity with task-positive networks, not the DMN+, further suggesting thalamic subnuclei can interface with preferred intrinsic networks and not only the DMN during the rest state (Ely et al., 2019). The present results provide evidence for a functional link between the PVT and DMN.

Notably, the PVT showed greater functional connectivity in DMN+ regions compared to the CeM and Pf (see activity in cool colors in Figure 6). The closest thalamic nucleus to PVT, that is structurally similar and of a similar size is the CeM. The CeM shares many connections with the PVT, but with additional projections to primary and secondary motor structures (Vertes et al., 2015). This is consistent with numerically stronger magnitude of functional connectivity of CeM compared to the other control regions’ plots (which tended to show a PVT > CeM > CL > Pf pattern). The CeM FC showed some correspondence with the DMN+ (although a numerically weaker match than PVT) as well at the somato-motor network (SOM; see Figure 5B).

Several recent frameworks have placed the PVT within the DMN. Given anterior thalamic lesion evidence of memory deficits, the anterior thalamic nuclei have been previously situated with the DMN in a network called the Posterior Medial System (Ranganath and Ritchey, 2012), a large-scale network associated with episodic recollection. The PVT-DMN functional connectivity is also consistent with a recent neuroanatomical model of the DMN that positions the anterior and mediodorsal thalamus with high centrality within the DMN (Alves et al., 2019). Intriguingly, Alves and colleagues also found that the VTA and NAc also fall within the DMN circuitry, further implicating the midbrain in the DMN, and suggest the anterior and mediodorsal thalamus facilitates integration across systems involved in memory, reward, and emotion. These prior studies also suggest that damage or dysfunction of the PVT-DMN link could induce DMN dysfunction found in neuropsychiatric diseases and memory disorders (Price and Drevets, 2012; Alves et al., 2019).

### Relevance to Neuropsychiatric Diseases in Humans

The PVT has been implicated in depressive-, anxiety-, and fear-like behaviors (Li et al., 2010a,b; Zhu et al., 2011; Hsu et al., 2014), suggesting dysfunction of the PVT might contribute to maladaptive behavior and mood states relevant to neuropsychiatric diseases (Do-Monte et al., 2016). For instance, the PVT is thought to play a mediating role between chronic stress and major depressive disorder (MDD; Hsu et al., 2014). Mitochondrial DNA deletions in the PVT, to a greater extent than the NAc IL, PL and amygdala, have been linked with depressive-episode-like behaviors in mice (Kasahara et al., 2016), again suggesting a PVT-centered circuit is important in mood regulation. The forced swim test in rodents, a model of depressive-like behaviors, was related to coactivation of the PVT and amygdala, along with the mPFC, BNST, and NAc (Zhu et al., 2011). In humans, MDD diagnostic status and symptom severity have been linked with increased medial thalamo–cortical functional connectivity (Brown et al., 2017). Dysfunction in the cortical-striatal-pallidal-thalamic loop—a circuit that includes the DMN and PVT—is thought to contribute to the pathophysiology of neuropsychiatric disorders (Price and Drevets, 2012). Given the role of the PVT in retrieving remote stress and fear memories and recall of fear extinction (Tao et al., 2020), the PVT might contribute to the circuit associated trauma memory-related symptoms of post-traumatic stress order (Yehuda and LeDoux, 2007; Do-Monte et al., 2016). The PVT is not only responsive to natural or intrinsic rewards, but also in drug-seeking behavior and addiction (Matzeu et al., 2014; Zhu et al., 2016; Keyes et al., 2020). Understanding the intrinsic connectivity of the PVT in healthy young adult humans provides a necessary foundation and reference point for future research assessing how dysfunction in this circuitry is related to the presence and severity of neuropsychiatric disease.

### Limitations and Future Directions

There are several limitations to our study and caveats to bear in mind when interpreting the present results. First, due to the atlas-construction process implemented by Krauth et al. (2010), there are important anatomical discrepancies between the final digital atlas PVT seed used in the current study and previous thalamic atlases. Consistent with previous atlases (e.g., Mai et al., 2004 and The Allen Brain Atlas)^7^, the initial reference mesh prior to the digitization process in Krauth et al. (2010) displays the PVT as a thin band lining the dorsomedial surface of the third ventricle, extending posteriorly from anterior dorsomedial area to the habenula (see Figure 3A in Krauth et al., 2010). However, due to the atlas-construction process, the final morphed PVT region appears biased toward the aPVT and possibly the anterior portion of surrounding subnuclei (see Figure 3B in Krauth et al., 2010). Thus, the current results might be biased toward aPVT FC. Nevertheless, the current findings appear to be sensitive to known connectivity of the pPVT (e.g., insula, BNST, amygdala, and prelimbic areas). Second, by definition, rsfMRI measures intrinsic functional connectivity during rest. This prevents us from assessing the crucial role of PVT and its projections during reward-related, emotional, and other tasks. Third, rsfMRI consists of a time series of data, and the current analyses quantified the correlation between regions across the entire timeseries, which reflects static or stationary functional connectivity. In the future, functional connectivity analyses focusing on dynamic changes in the PVT network configuration across the timeseries could reveal meaningful patterns. For instance, it is possible that the PVT also shows strong but relatively intermittent network connectivity with other nodes of the reward, limbic, or salience networks. Fourth, we compared PVT connectivity to other thalamic regions which we considered controls. Yet, these nuclei may have shared connectivity with the PVT, which will be obscured by this type of analysis. Moreover, the size difference between the subnuclei control regions and the smaller PVT seed might lead to temporal signal-to-noise differences that could bias the whole-brain PVT FC comparisons reported in Figure 6. Thus, the whole-brain comparisons should be interpreted with caution. Temporal signal-to-noise ratios between the seed could lead to noise and sensitivity issues that could result in false positive or false negatives, respectively. Fifth, it is well-known that smoothing kernel size can influence group-level results, thus, it is not clear how the patterns might change as a function of kernel size or if the data were not spatially smoothed. However, we chose to smooth the dataset in order to better bridge the 7T and 3T findings and because it was not our primary aim to map intra-thalamic connectivity. Additionally, one should note that we smoothed in volumetric space, which, unlike cortical surface smoothing (i.e., CIFTI), might smooth across sulci and tissue compartments (Coalson et al., 2018). Finally, the current resolution can only speak to connections of the PVT as whole, and not the complex structural and functional connectivity along the anterior-posterior extent of the PVT shown in rodents and non-human primates.

The current work used a large sample of high-quality, long-duration fMRI data from The Human Connectome Project to map the resting FC of the PVT at the highest available 7T resolution with the aim of bridging the work for investigators using 3T MRI scanners. For future 3T fMRI work using the PVT seed from Krauth et al. (2010) and Jakab et al. (2012), we recommend using the 3D mask that detected significant levels of FC by both magnet strengths [mask available in the Open Science Framework (OSF) repository: https://osf.io/re3v6/].

Future work will capitalize on this study to determine PVT functional connectivity separately in men and women. The developmental profile of PVT connectivity, as well as changes with aging, requires examination. Importantly, establishment of global or specific alterations of the PVT connectivity map in the context of affective and cognitive disorders should be a major focus of future work, and will strongly benefit from the normative data provided here.

## Conclusions

The thalamus is classically known for its role in relaying sensory information, but accumulating evidence in animals and humans converge on discrete roles of the subnuclei of the midline thalamus (Van der Werf et al., 2002; Kirouac, 2015; Vertes et al., 2015) and a complex and integrative role for the PVT. The PVT has been implicated in stress, memory, motivated functions and behaviors, and mood states (Hsu et al., 2014; Kirouac, 2015; Barson et al., 2020; McGinty and Otis, 2020). Do Monte and colleagues propose that the PVT is anatomically well-positioned to integrate aversive memory signals with adaptive biological responses in coordination with arousal, defensive and motivational behaviors, with stress and circadian rhythms signals (Do-Monte et al., 2016). Here, we provide evidence of significant alignment of the connectivity of the human PVT with that established for experimental models. The current work provides a requisite foundation for ongoing and future work in humans investigating the role of the human PVT in normal and pathological behaviors.

## Data Availability Statement

The datasets presented in this study can be found in online repositories. The names of the repository/repositories and accession number(s) can be found below: The 3D results Nifti file masks generated for this study can be found in the Open Science Framework (OSF) at: https://osf.io/re3v6/. Additional information not found in the OSF repository can be obtained by reasonable request to the corresponding author.

## Ethics Statement

The study was approved by Washington University in the St. Louis’ Human Research Protection Office (IRB #201204036). Written informed consent was obtained from all study participants. No study activities or procedures with human subjects took place at the authors’ institution. The current secondary analysis of the HCP data was deemed exempt from review by the Institutional Review Board of University of California, Irvine. All data were de-identified by HCP before public release and all HCP participants provided written informed consent to study procedures and data sharing outlined by HCP.

## Author Contributions

SMK, TZB, and MAY conceived the design of the study. SMK processed and analyzed the data. SMK and MAY interpreted the results. SMK wrote the article with input from TZB, MTB, and MAY who provided critical revision and feedback. All authors contributed to the article and approved the submitted version.

## Conflict of Interest

The authors declare that the research was conducted in the absence of any commercial or financial relationships that could be construed as a potential conflict of interest.

## Footnotes

^1^https://wiki.humanconnectome.org/

^2^http://www.nitrc.org/projects/conn

^3^http://people.cas.sc.edu/rorden/mricron/index.html

^4^http://www.mccauslandcenter.sc.edu/mricrogl/home

^5^http://www.nitrc.org/projects/bnv/

^6^https://github.com/ejcorn/brain_states

^7^www.brain-map.org
