# Corrigendum: Functional Connectivity of the Human Paraventricular Thalamic Nucleus: Insights From High Field Functional MRI

**DOI:** 10.3389/fnint.2021.724071

**Published:** 2021-07-26

**Authors:** Sarah M. Kark, Matthew T. Birnie, Tallie Z. Baram, Michael A. Yassa

**Affiliations:** ^1^Center for the Neurobiology of Learning and Memory, University of California, Irvine, Irvine, CA, United States; ^2^Department of Neurobiology and Behavior, University of California, Irvine, Irvine, CA, United States; ^3^Department of Pediatrics, University of California, Irvine, Irvine, CA, United States; ^4^Department of Anatomy & Neurobiology, University of California, Irvine, Irvine, CA, United States

**Keywords:** paraventricular thalamic nucleus, reward, resting state functional connectivity, brain circuit, neuroimaging

In the original article, there were a couple of errors that do not alter the results or conclusions.

In the original article, there was an error. The motion artifact thresholds were set to framewise motion > 0.5 mm (not ≥ 0.3 mm) or Global Signal Change *z* > 3 (not *z* ≥ 3).

A correction has been made to ***Materials and Methods, MRI Analysis, Preprocessing and De-Noising, Paragraph 3:***

“Artifact identification was performed using Artifact Detection Tools (ART) implemented in CONN. We enforced conservative motion censoring thresholds, scrubbing frames exceeding > 0.5 mm frame-wise motion or Global Signal Change *z* > 3. At this stage, 14 of the 135 participants were dropped from the 7T analyses for having fewer valid scans than the rest of the group (as defined by 1st Q – 1.5 IQR; see **Supplementary Figure 1**). Thus, the final sample to be considered in 2nd-level analysis consisted of 121 participants.”

In the original article, there was an error. There were typos in the **Figure 1** caption: “Thalamus seeds” has been replaced by “Thalamus seed” and “CeM (blue)” has been replaced by “CeM (green).”

A correction has been made to the ***Figure 1 caption***:

“Thalamus seed regions entered into functional connectivity analyses. **(A)** The Paraventricular Thalamic Nucleus (PVT) seed (shown in yellow) surrounded by the rest of the thalamus (shown in purple). The purple seed region encompassing the rest of the thalamus was used for the analyses presented in **Figures 2–4**. **(B)** In separate analyses, we also compared PVT functional connectivity with nearby midline thalamic subnuclei CeM (green), CL (red), and the Pf (blue; see **Figures 5, 6**).”

In the original article, there was an error. The 7T contrasts comparing PVT functional connectivity to the control subnuclei used semi-partial maps, not bivariate maps.

A correction has been made to ***Materials and Methods, Group-Level Analysis and Thresholding, PVT Functional Connectivity Compared to Other Midline Thalamic Subnuclei, Paragraph 1:***

“Controlling for the average signal from the remainder of the thalamus (i.e., thalamus without the PVT mask) using semi-partial correlations demarcated unique functional connectivity while controlling for average thalamic signal. However, to reveal functional connectivity that was stronger for the PVT compared to other midline areas we conducted further analyses comparing semi-partial PVT correlations individually to other nearby medial subnuclei (CeM, CL, and Pf, see **Figure 1B**). For instance, we tested the conjunction of the thresholded SBC maps using SPM12 *imcalc* (e.g., PVT bivariate map ⋂ PVT > CL semi-partial map both thresholded separately at *p*TFCE-FWE < 0.05). We conjoined the maps to ensure activity differences were not driven by a strong negative correlation with the comparison region. We also conducted a supplementary whole-brain pairwise comparison of bivariate connectivity that was significantly greater for the PVT compared to the rest of the thalamus (i.e., PVT functional connectivity [bivariate] ⋂ PVT > Rest of the thalamus). It is important to note that there are likely substantially different temporal signal-to-noise ratios for the signal measured from 14 cubic mm PVT voxels compared to the other subnuclei.”

A correction has been made to ***Results, 7T Dataset, Direct Comparison of PVT Functional Connectivity to Control Seeds, Paragraph 1:***

“Thus far, the results report on significant PVT functional connectivity that survives controlling for functional connectivity of the control regions and represents unique functional connectivity. We next tested where PVT functional connectivity exceeded that of the control regions. Whole-brain comparisons of the semi-partial correlations for the PVT compared to the CeM, CL, and Pf, separately, showed greater positive PVT functional connectivity with the amygdala, hippocampus, ventromedial PFC/orbital frontal gyrus (Brodmann area 11) and dorsomedial PFC (Brodmann area 9 and 10), left lateral orbital frontal gyrus (Brodmann area 47), and middle temporal gyrus/temporal pole (see overlap of the three maps shown in white [left] and corresponding raincloud plots [right] in **Figure 6**). Voxels in the NAc were found in the maps of PVT > Pf and to a lesser extent PVT > CeM, but not PVT > CL.”

A correction has been made to ***the caption of Figure 6:***

“**(A)** Regions that show positive PVT functional connectivity that is greater than functional connectivity of the CeM (green), CL (red), or Pf subnuclei (blue; e.g., PVT bivariate map ⋂ PVT > CL semi-partial map both thresholded separately at *p*TFCE-FWE < 0.05). Some areas showed greater functional connectivity compared to CL and CeM (yellow), CeM and Pf (cyan), CL and Pf (violet), or all three (white). **(B)** Raincloud plots display density plots with the individual Fisher *r*-to-*Z* connectivity values for each participant extracted from regions that showed overlap between all three maps. Functional connectivity estimates from the separate ROIs are displayed in colors corresponding to the legend at the bottom. From top-to-bottom the subplots are displayed in descending order based on average effect size as calculated by paired Cohen's d. Inset color mixing image (lower left) accessed from https://colourware.wordpress.com/ (colourmixing.gif, 2013).”

In the original article, there was an error. The 3T seed data were drawn from the smoothed maps, not the unsmoothed maps.

A correction has been made to ***Materials and Methods, MRI Analysis, Preprocessing and De-Noising, Paragraph 4:***

“The functional data were further denoised using the CONN Toolbox's *aCompCor* method (white matter and CSF noise, frame-wise motion regression, artifact scrubbing, and linear detrending). The data were then band-pass filtered to isolate resting-state frequencies (0.01 Hz < *f* < 0.10 Hz) using a fast Fourier transform (FFT). Importantly, the 7T seed region of interest BOLD timeseries were extracted from resulting unsmoothed and denoised rsfMRI volumes to prevent “spillover” from other nearby regions. For the 3T dataset, masks were applied to smoothed data, which have higher signal-to-noise ratio. Denoising was successful in this final sample, eliminating the positive skew and inter-subject variability related to physiological and motion artifact in randomly sampled functional connectivity (**Supplementary Figure 2**) and there was no evidence for a link between mean motion or mean global signal change and functional connectivity in the 7T or 3T datasets (see **Supplementary Figures 3, 4**).”

A correction has been made to ***Materials and Methods, MRI Analysis, Seed Regions, Paragraph 2:***

“To avoid the loss of small ROI information in the 1.6 and 2 mm functional spaces, the CONN Toolbox whole-brain seed-to-voxel analysis extracts and averages the BOLD timeseries from the closest corresponding voxels (e.g., 14 total voxels for the PVT) in the functional maps to correlate with the non-PVT voxels in the smoothed maps. This approach, as opposed to first resampling the small ROI subnuclei, guarantees an appropriate partial-volume weighting of the functional data and no loss of smaller ROIs like the PVT or the subnuclei.”

In the original article, there was a mistake in Supplementary Figures 9, 10 as published. The figures depicted were missing the 3T Negative FC map (blue mask). The updated figures are shown below. The **Supplementary Material** document has been updated.

**Figure 9:**
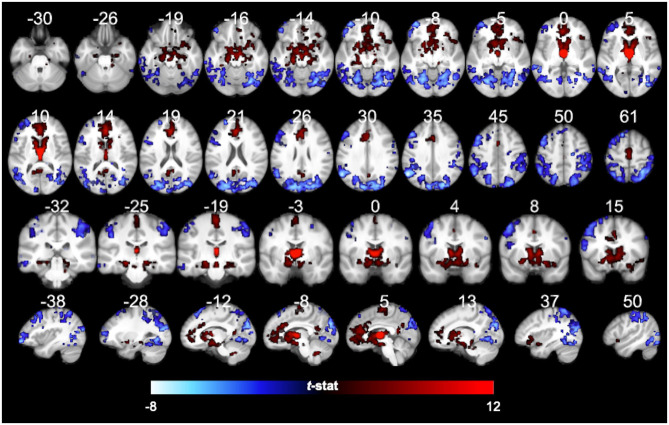


**Figure 10:**
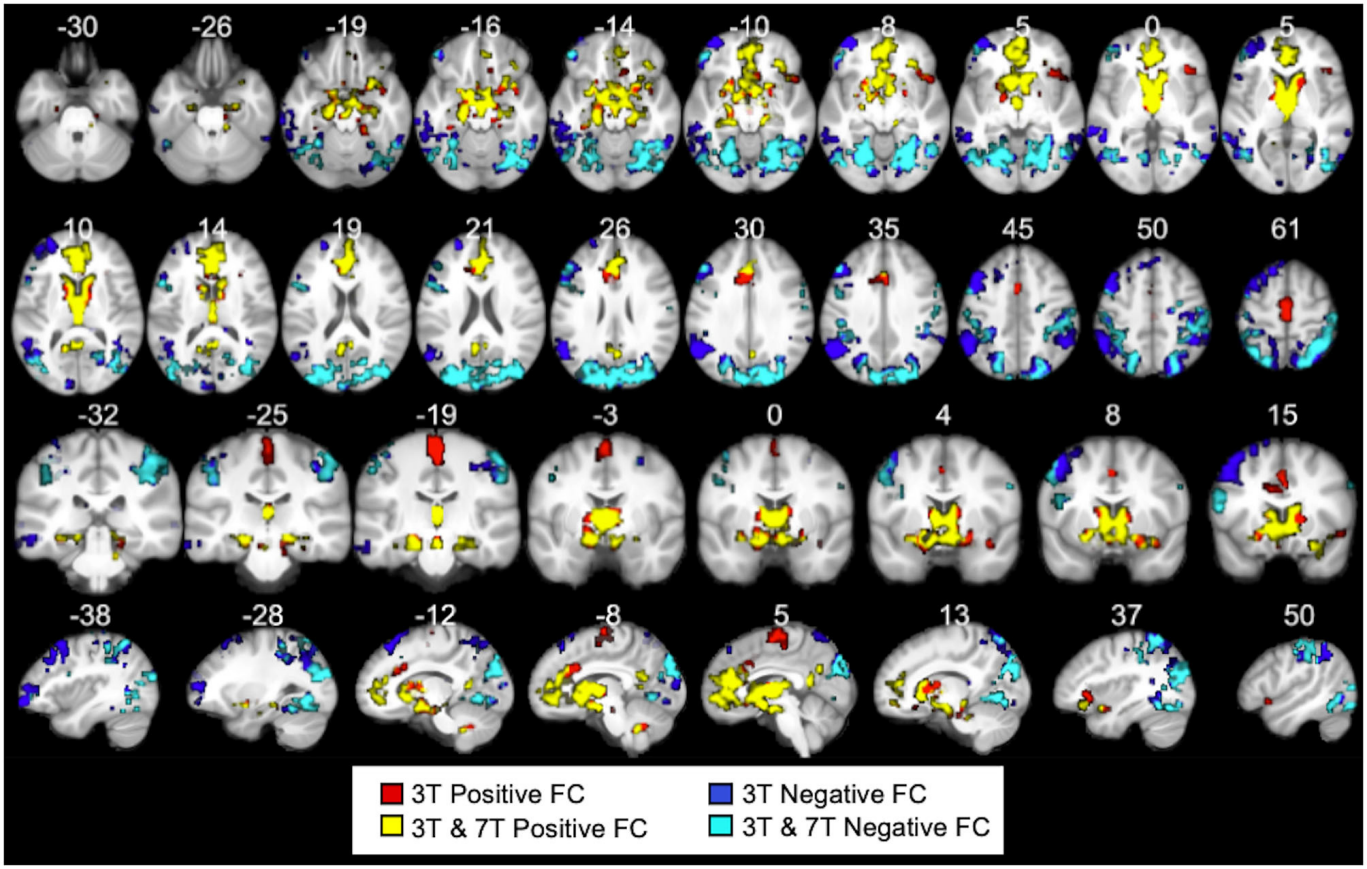


The authors apologize for these errors and state that they do not change the scientific conclusions of the article in any way. The original article has been updated.

## Publisher's Note

All claims expressed in this article are solely those of the authors and do not necessarily represent those of their affiliated organizations, or those of the publisher, the editors and the reviewers. Any product that may be evaluated in this article, or claim that may be made by its manufacturer, is not guaranteed or endorsed by the publisher.

