# Supplementary material for: Functional Connectivity of the Human Paraventricular Thalamic Nucleus: Insights From High Field Functional MRI
Source: Front Integr Neurosci. 2021 Apr 21;15:662293. doi: 10.3389/fnint.2021.662293 (PMC8096909; doi:10.3389/fnint.2021.662293)
Supplement: Supplementary file 1 [file Data_Sheet_1.pdf]

Supplementary Material

Functional connectivity of the human paraventricular thalamic nucleus: results from high field magnetic resonance imaging

Supplementary Table 1. Clusters identified by TCFE of the bivariate Pvt results before overlapping with the semi-partial maps that control for connectivity with other regions of the thalamus. Clusters in this table correspond to the clusters shown in Supplementary Figure 1. FEW=Family-wise error rate, FC=functional connectivity, FDR=False-discovery rate, MNI=Montreal Neurological Institute, TFCE=Threshold-free Cluster enhancement.

Cluster Index	FC Association	Cluster Peak MNI coordinates (x , y, z)			voxel extent	peaks	TFCE	peak p-FWE	peak p-FDR	peak p-uncorr
1	Positive	-1	-9	-2	133633	1406	1752203.28	0	0	0
2	Negative	-12	-84	27	73933	1415	5755.46	0	0	0
3	Positive	-40	-75	27	2061	13	2016.58	0	0	0
4	Positive	60	-52	22	2235	17	1566.94	0	0	0
5	Negative	-44	50	-16	1649	45	1175.89	0.006	0.000013	0.000007

Supplementary Table 2. Coverage of regions of the The Human Brainnetome (BN) Atlas that show significant positive FC of the Pvt, controlling for signal from the rest of the thalamus (corresponds to activity shown in red in Figure 2 and 3). For more information on cyto-architectonic labeling see the guide to the The Human Brainnetome (BN) Atlas in Fan et al., 2016. Lighter red shading in the voxel extent and % voxels columns signifies more coverage. Table limited to regions with 10 or more suprathreshold voxels. BN ID=reference ID for cluster number in BN atlas, Hem=hemisphere, L=left, R=right. *Pvt mask falls within this thalamic region of the BN atlas.

Lobe	Gyrus	Hem	Modified cyto-architectonic	BN ID	voxel extent	% voxels
Frontal	Middle Frontal Gyrus	L	dorsal area 9/46	15	331	0.211
		L	area 46	19	177	0.165
		R	dorsal area 9/46	16	197	0.137
		R	lateral area 10	28	145	0.099
		L	lateral area 10	27	100	0.091
		L	ventrolateral area 8	23	49	0.037
		R	area 46	20	23	0.016
	Orbital Gyrus	L	medial area 14	41	589	0.975
		R	medial area 14	42	835	0.951
		L	orbital area 12/47	43	291	0.505
		R	orbital area 12/47	44	255	0.451

		L	area 13	49	363	0.320
		R	medial area 11	48	265	0.279
		L	medial area 11	47	151	0.201
		R	lateral area 12/47	52	89	0.150
		R	lateral area 11	46	245	0.149
		R	area 13	50	110	0.111
		L	lateral area 11	45	121	0.093
		L	lateral area 12/47	51	63	0.092
	Superior Frontal Gyrus	L	medial area 10	13	1371	1.000
		R	medial area 10	14	1312	0.992
		L	lateral area 9	5	560	0.619
		R	medial area 9	12	642	0.542
		R	lateral area 9	6	583	0.520
		L	medial area 9	11	287	0.359
		R	dorsolateral area 8	4	283	0.313
		L	dorsolateral area 8	3	292	0.254
Insular	Ventral Anterior Insula	L	ventral agranular insula	165	260	0.949
		R	ventral agranular insula	166	206	0.858
	Ventral Posterior Insula	L	ventral granular insula	169	10	0.030
Limbic	Cingulate Gyrus	L	dorsal area 23	175	541	1.000
		R	subgenual area 32	188	516	1.000
		R	dorsal area 23	176	497	0.996
		L	subgenual area 32	187	767	0.977
		L	ventral area 23	181	335	0.866
		R	ventral area 23	182	269	0.865
		R	rostroventral area 24	178	200	0.855
		L	pregenual area 32	179	459	0.738
		L	rostroventral area 24	177	70	0.476
		R	pregenual area 32	180	215	0.462
		R	caudal area 23	186	216	0.315
		L	caudal area 23	185	163	0.207
Occipital	Cuneus	R	ventromedial parieto-occipital sulcus	198	45	0.037
		L	ventromedial parieto-occipital sulcus	197	15	0.012
		R	rostral cuneus gyrus	192	11	0.010
Parietal	Angular Gyrus	L	rostroventral area 39 (PGa)	143	1113	0.489
		R	rostroventral area 39 (PGa)	144	914	0.459
		L	rostrodorsal area 39 (Hip3)	137	74	0.069
		R	rostrodorsal area 39 (Hip3)	138	78	0.058

		R	caudal area 39 (PGp)	136	33	0.020
	Precuneus	L	area 31 (Lc1)	153	960	0.988
		R	area 31 (Lc1)	154	1159	0.905
		L	dorsomedial parietooccipital sulcus (PEr)	151	791	0.728
		R	dorsomedial parietooccipital sulcus (PEr)	152	701	0.499
		L	medial area 7 (PEp)	147	184	0.297
		R	medial area 7 (PEp)	148	76	0.133
Subcortical Nuclei	Amygdala	L	medial amygdala	211	50	0.327
		L	lateral amygdala	213	12	0.179
		R	lateral amygdala	214	10	0.104
		R	medial amygdala	212	22	0.099
	Hippocampus	L	caudal hippocampus	217	560	0.832
		R	caudal hippocampus	218	552	0.730
		L	rostral hippocampus	215	397	0.573
		R	rostral hippocampus	216	283	0.465
	Striatum	L	ventral caudate	219	420	0.791
		R	nucleus accumbens	224	280	0.698
		R	ventral caudate	220	226	0.657
		L	nucleus accumbens	223	172	0.608
		R	dorsal caudate	228	381	0.436
		L	dorsal caudate	227	226	0.390
		L	ventromedial putamen	225	27	0.077
	Thalamus	R	rostral temporal thalamus*	238	197	0.952
		L	rostral temporal thalamus*	237	91	0.892
		L	medial pre-frontal thalamus	231	119	0.578
		R	medial pre-frontal thalamus	232	86	0.562
		R	occipital thalamus	242	21	0.226
		R	caudal temporal thalamus	244	28	0.220
		L	caudal temporal thalamus	243	19	0.147
		R	lateral pre-frontal thalamus	246	19	0.082
		L	lateral pre-frontal thalamus	245	27	0.072
Temporal	Fusiform Gyrus	L	rostroventral area 20	103	29	0.023
		R	rostroventral area 20	104	20	0.015
		R	lateroventral area 37	108	10	0.009
	Inferior Temporal Gyrus	L	intermediate lateral area 20	95	41	0.085
		R	intermediate lateral area 20	96	29	0.053
		L	rostral area 20	93	16	0.025
	Middle Temporal Gyrus	R	rostral area 21	84	782	0.638

		L	anterior superior temporal sulcus	87	646	0.616
		R	anterior superior temporal sulcus	88	845	0.523
		L	rostral area 21	83	483	0.497
		L	caudal area 21	81	216	0.321
		R	caudal area 21	82	146	0.171
	Parahippocampal Gyrus	R	area 28/34 (EC, entorhinal cortex)	116	77	0.626
		L	area 28/34 (EC, entorhinal cortex)	115	87	0.580
		R	area TL (lateral PPHC, posterior parahippocampal gyrus)	114	31	0.290
		L	area TL (lateral PPHC, posterior parahippocampal gyrus)	113	37	0.270
		R	caudal area 35/36	112	41	0.232
		L	area TH (medial PPHC)	119	27	0.221
		L	area TI (temporal agranular insular cortex)	117	13	0.122
		L	caudal area 35/36	111	10	0.073
	Superior Temporal Gyrus	R	medial area 38	70	441	0.529
		R	lateral area 38	78	178	0.236
		L	medial area 38	69	176	0.189
		L	lateral area 38	77	87	0.155
		L	rostral area 22	79	44	0.061
		R	rostral area 22	80	14	0.054

Supplementary Table 3. Coverage of regions of the The Human Brainnetome (BN) Atlas that show significant negative FC of the Pvt, controlling for signal from the rest of the thalamus (corresponds to activity shown in blue in Figure 2 and 3). For more information on cyto-architectonic labeling see the guide to the The Human Brainnetome (BN) Atlas in Fan et al., 2016. Lighter blue shading in the voxel extent and % voxels columns signifies more coverage. Table limited to regions with 10 or more suprathreshold voxels. BN ID=reference ID for cluster number in BN atlas, Hem=hemisphere, L=left, R=right.

**Lobe**	**Gyrus**	**Hem**	**Modified cyto-architectonic**	**BN ID**	**voxel extent**	**% voxels**
Frontal Lobe	Inferior Frontal Gyrus	L	dorsal area 44	29	160	0.447
		L	inferior frontal sulcus	31	176	0.381
		L	ventro area 44	39	123	0.353
		L	rostral area 45	35	159	0.335

		R	ventro area 44	40	100	0.281
		R	rostral area 45	36	75	0.135
		R	dorsal area 44	30	35	0.083
	Middle Frontal Gyrus	R	lateral area10	28	200	0.136
		L	ventrolateral area 6	25	82	0.104
		L	ventro area 9/46	21	122	0.090
		R	ventro area 9/46	22	109	0.085
		R	ventrolateral area 6	26	20	0.027
		L	inferior frontal junction	17	21	0.021
	Orbital Gyrus	R	orbital area 12/47	44	165	0.292
		L	lateral area 12/47	51	127	0.185
		R	lateral area 12/47	52	71	0.120
		L	orbital area 12/47	43	47	0.082
	Precentral Gyrus	R	area 4 (head and face region)	54	503	0.911
		R	caudal ventrolateral area 6	64	744	0.821
		L	caudal ventrolateral area 6	63	702	0.743
		L	area 4 (head and face region)	53	549	0.531
		R	caudal dorsolateral area 6	56	575	0.481
		R	area 4 (tongue and larynx region)	62	145	0.337
		L	caudal dorsolateral area 6	55	290	0.320
		L	area 4 (tongue and larynx region)	61	95	0.192
		R	area 4 (upper limb region)	58	100	0.156
		L	area 4 (upper limb region)	57	70	0.092
	Superior Frontal Gyrus	L	dorsolateral area 6	7	98	0.113
		R	dorsolateral area 6	8	76	0.098
Insular Lobe	Caudoventro Anterior Insula	R	dorsal dysgranular insula	174	83	0.266
		L	dorsal dysgranular insula	173	24	0.049
	Rostrodorsal Posterior Insula	L	dorsal granular insula	171	34	0.096
		R	dorsal granular insula	172	22	0.074
Occipital Lobe	Cuneus	R	rostral lingual gyrus	196	1041	0.920
		L	rostral lingual gyrus	195	809	0.854
		R	caudal cuneus gyrus	194	190	0.264
		L	caudal lingual gyrus	189	173	0.249
		L	rostral cuneus gyrus	191	263	0.244
		L	ventromedial parietooccipital sulcus	197	258	0.210

		R	ventromedial parietooccipital sulcus	198	203	0.168
		R	caudal lingual gyrus	190	111	0.137
		R	rostral cuneus gyrus	192	126	0.114
		L	caudal cuneus gyrus	193	13	0.016
	Occipital Gyrus	L	middle occipital gyrus	199	846	0.813
		L	area V5/MT	201	767	0.735
		R	area V5/MT	202	702	0.634
		R	middle occipital gyrus	200	636	0.576
		L	inferior occipital gyrus	205	333	0.235
		R	inferior occipital gyrus	206	254	0.199
		R	occipital polar cortex	204	140	0.106
		L	occipital polar cortex	203	81	0.063
	Superior Occipital Gyrus	L	medial superior occipital gyrus	207	652	0.921
		L	lateral superior occipital gyrus	209	597	0.870
		R	medial superior occipital gyrus	208	693	0.831
		R	lateral superior occipital gyrus	210	748	0.732
	Angular Gyrus	L	caudal area 39 (PGp)	135	543	0.400
		R	caudal area 39 (PGp)	136	604	0.369
		R	rostrodorsal area 39 (Hip3)	138	44	0.033
		L	rostroventro area 39 (PGa)	143	31	0.014
	Postcentral Gyrus	L	area 2	159	697	0.774
		L	area 1/2/3 (upper limb, head and face region)	155	741	0.731
		R	area 2	160	555	0.666
		R	area 1/2/3 (upper limb, head and face region)	156	575	0.546
		R	area 1/2/3 (tongue and larynx region)	158	394	0.499
		L	area 1/2/3 (tongue and larynx region)	157	327	0.426
		R	area1/2/3 (trunk region)	162	12	0.022
	Precuneus	R	dorsomedial parietooccipital sulcus (PEr)	152	162	0.115
		L	dorsomedial parietooccipital sulcus (PEr)	151	29	0.027
	Superior Parietal Lobule	L	rostral area 7	133	412	0.800
		R	rostral area 7	134	410	0.756
		R	postcentral area 7	130	315	0.714
		L	postcentral area 7	129	383	0.651
		R	caudal area 7	128	299	0.466

		L	intraparietal area 7 (hIP3)	125	189	0.391
		L	caudal area 7	127	229	0.389
		R	intraparietal area 7 (hIP3)	126	205	0.313
		L	lateral area 5	131	37	0.065
	Supramarginal Gyrus	R	rostrodorsal area 40 (PFt)	140	839	0.616
		L	rostrodorsal area 40 (PFt)	139	613	0.466
		R	rostroventro area 40 (PFop)	146	170	0.102
		R	caudal area 40 (PFm)	142	25	0.015
		L	rostroventro area 40 (PFop)	145	19	0.013
Temporal Lobe	Fusiform Gyrus	L	medioventro area37	105	940	0.880
		R	medioventro area37	106	804	0.860
		R	lateroventro area37	108	439	0.381
		L	lateroventro area37	107	464	0.354
	Inferior Temporal Gyrus	R	ventrolateral area 37	98	218	0.532
		R	extreme lateroventro area 37	92	121	0.467
		L	extreme lateroventro area 37	91	67	0.208
		L	ventrolateral area 37	97	98	0.181
		L	intermediate ventro area 20	89	10	0.036
	Middle Temporal Gyrus	L	dorsolateral area37	85	38	0.054
		R	dorsolateral area37	86	35	0.037
	Posterior Superior Temporal Sulcus	R	caudoposterior superior temporal sulcus	124	35	0.108
	Superior Temporal Gyrus	R	TE1.0 and TE1.2	74	191	0.229
		L	TE1.0 and TE1.2	73	163	0.187
		R	caudal area 22	76	125	0.186
		L	caudal area 22	75	102	0.154
		L	area 41/42	71	61	0.120
		R	area 41/42	72	32	0.079
		R	rostral area 22	80	14	0.054
		L	lateral area 38	77	13	0.023
		R	lateral area 38	78	13	0.017

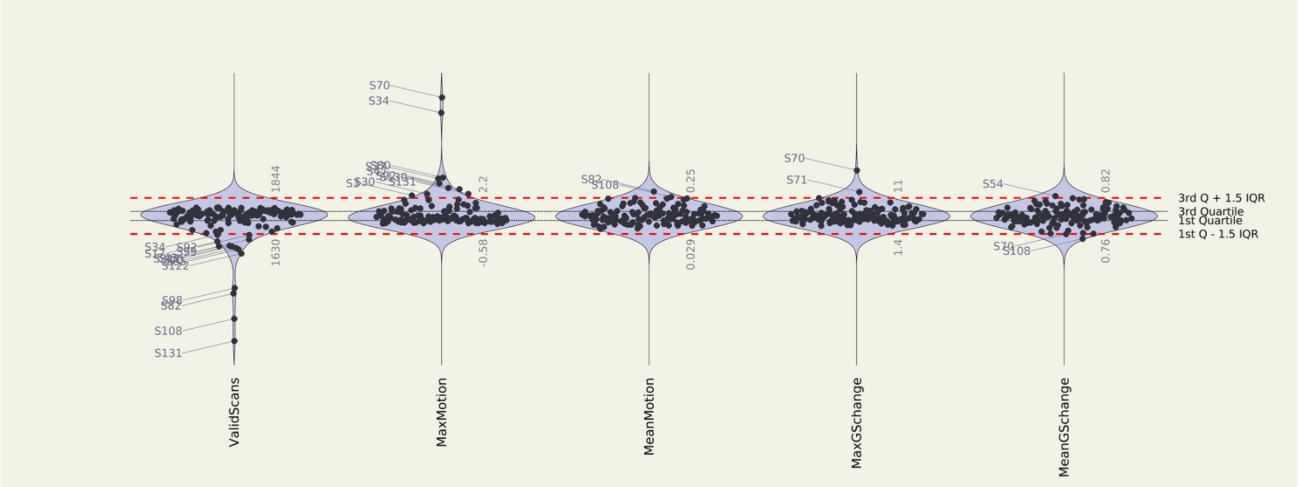

Supplementary Figure 1. Distribution of subject-level quality control measures for 135 participants originally entered into the 7T dataset. Participants just below the 1st Q – 1.5 IQR for ValidScans or above the 3rd Q + 1.5 IQR for MeanMotion were excluded from analyses (n=14).

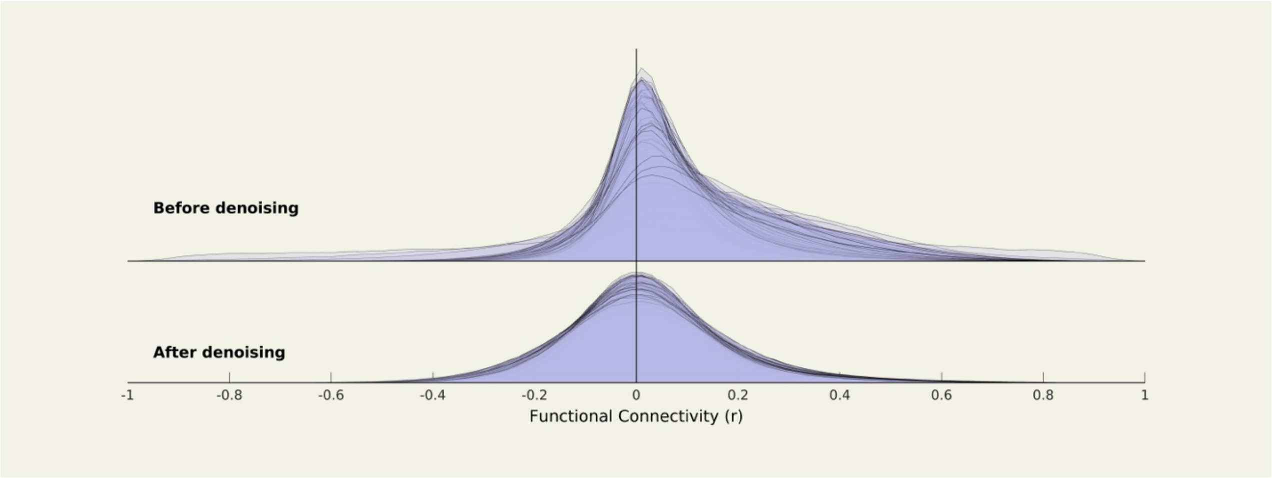

Supplementary Figure 2. Distribution of 1000 randomly sampled functional connectivity values before denoising (top) and after denoising (bottom) for 121 participants of the 7T used (excluding ValidScan outliers shown in Supplementary Figure 1). Before denoising there was relatively more positively-skew, consistent with physiological and motion artifact. After denoising the distributions exhibit less inter-subject variability and are less right-skewed.

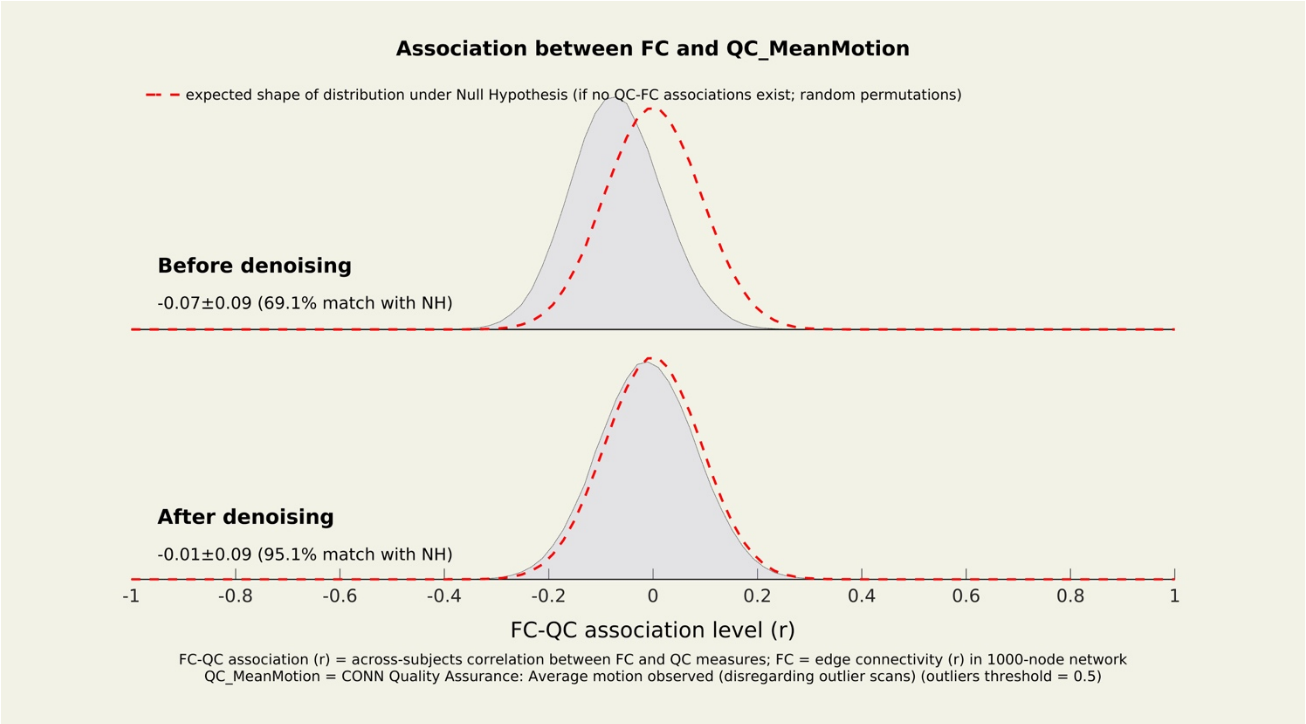

Supplementary Figure 3. Association between 7T FC and QC_MeanMotion for n=121. Representative figure showing the distribution of across subjection correlations between FC and Valid Scan. 95% match with the null hypothesis suggest a lack of relationship between motion QC and FC. Null hypothesis also matched for QC_ValidScans (number of useable TRs) and QC_MeanGSChange (mean global signal change across TRs).

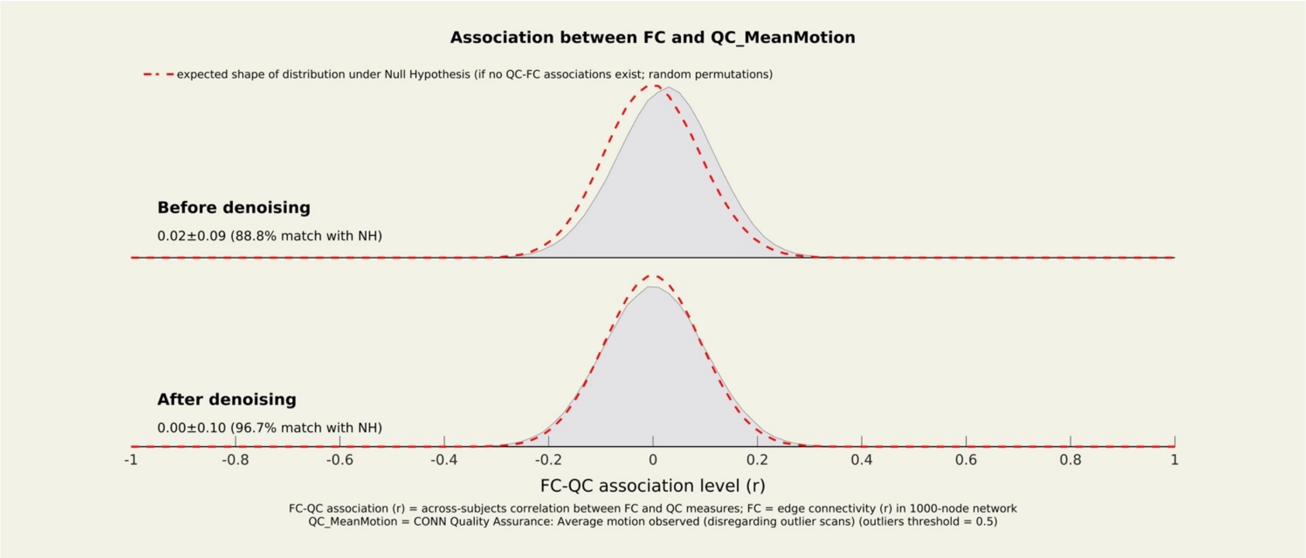

Supplementary Figure 4. Association between 3T FC and QC_MeanMotion n=121. Representative figure showing the distribution of across-subject correlations between FC and Mean Motion. 95% match with the null hypothesis suggest a lack of relationship between motion QC and FC. Null hypothesis also matched for QC_MeanGSChange (96.6%) but only a 90% match with the distribution for null for QC_ValidScans.

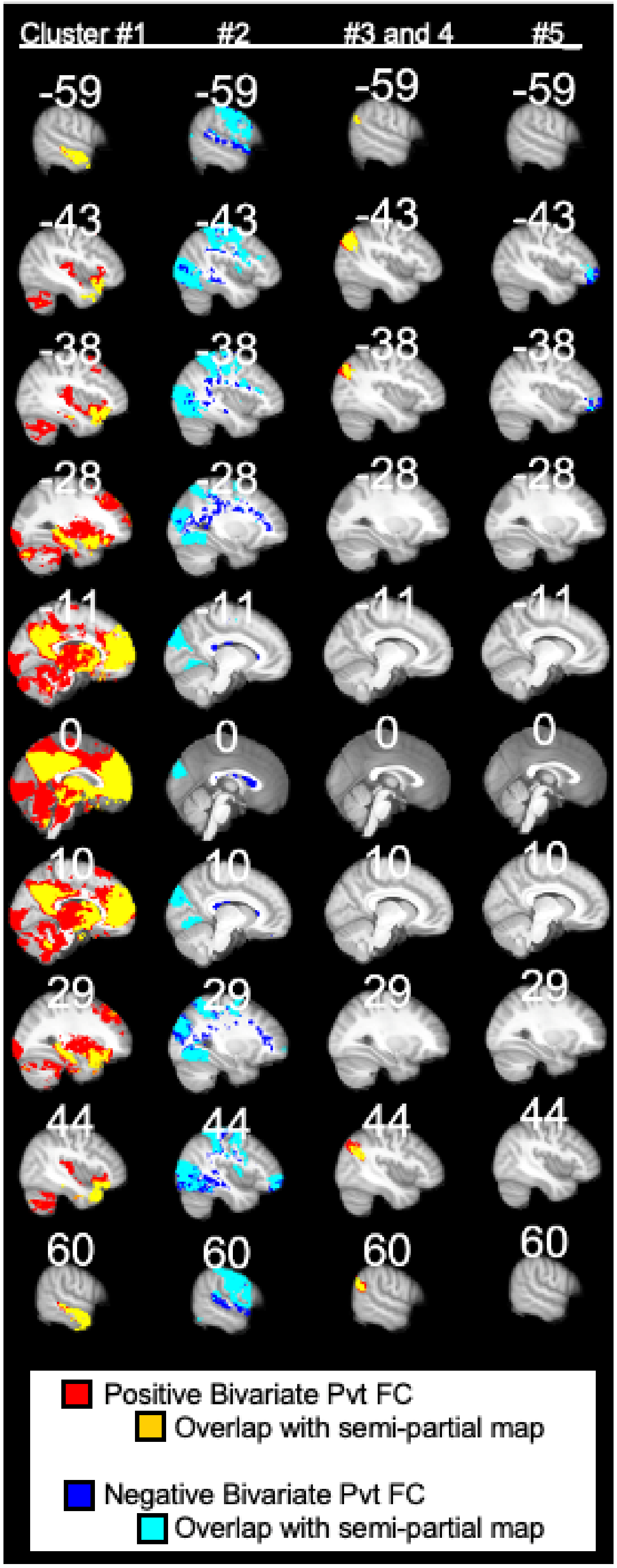

Supplementary Figure 5. Bivariate Pvt functional connectivity 7T results for Positive FC (red) and Negative FC (blue) before assessing the overlap with the semi-partial correlation maps that controls for signal from the rest of the thalamus (shown in yellow and cyan). All overlays thresholded at *p*TFCE-FWE<0.05.

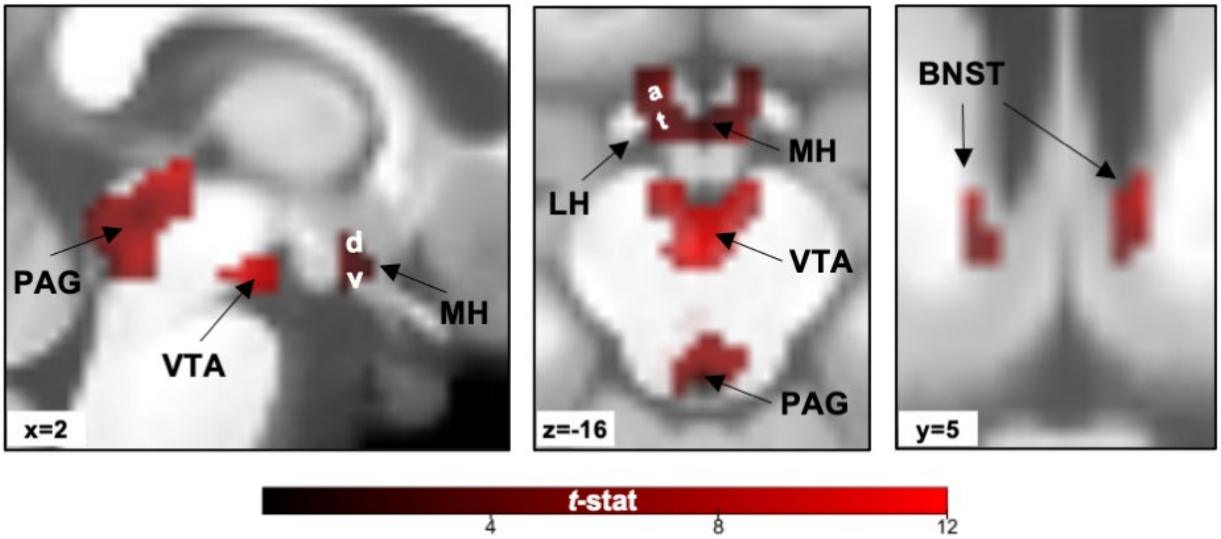

Supplementary Figure 6. Voxels that show Positive FC with the Pvt (controlling for the rest of the thalamus) within atlas masks of the bed nucleus of the stria terminalis (BNST), hypothalamus sub-areas investigated, periaqueductal gray (PAG), and the ventral tegmental area (VTA). Abbreviations: a=anterior, d=dorsal, BNST= Bed Nucleus of the Stria Terminalis, LHA= lateral hypothalamus, MH=medial hypothalamus, p=posterior, PAG=Periaqueductal Gray, t=tuberal, v=ventral, VTA=Ventral Tegmental Area.

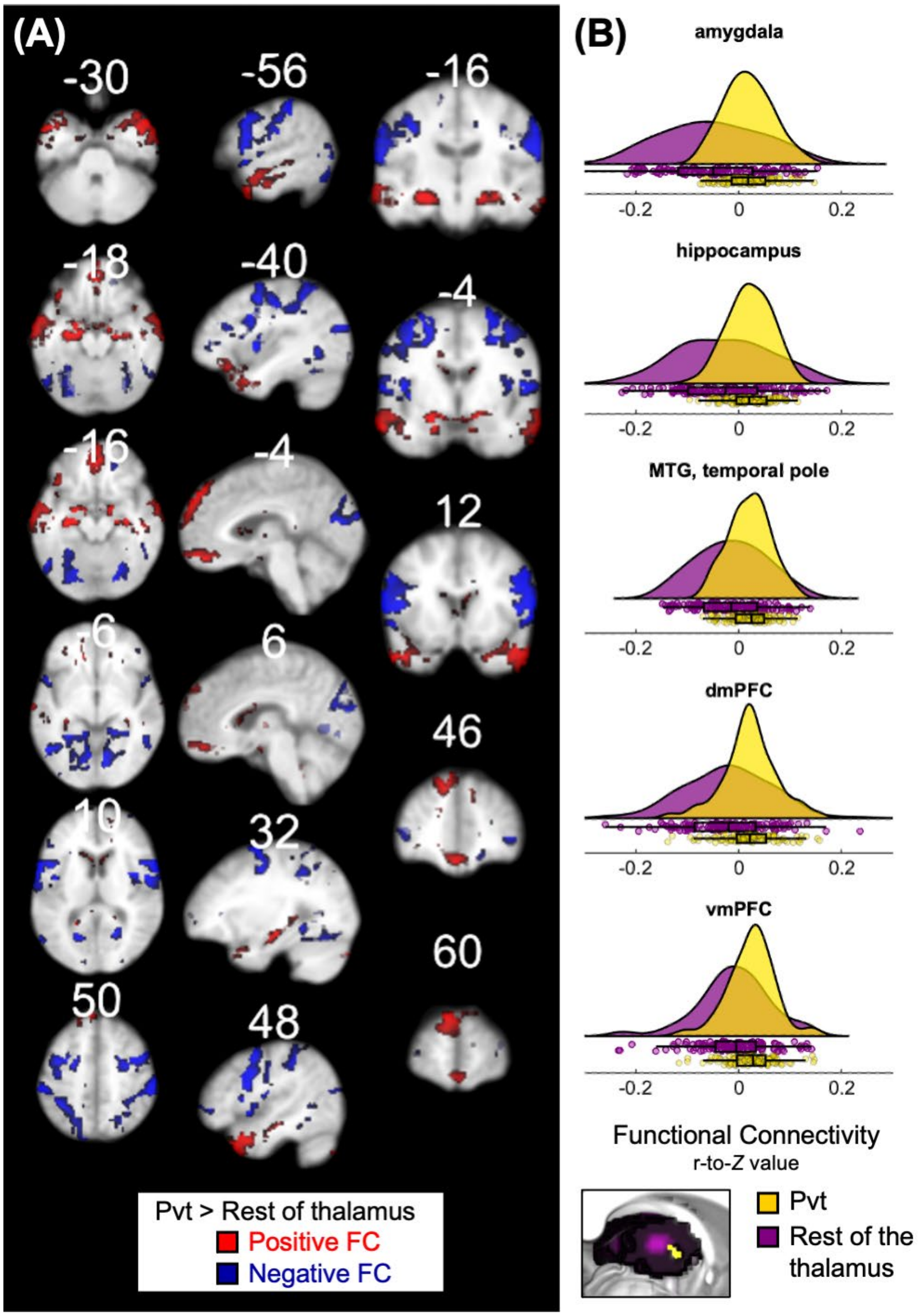

Supplementary Figure 7. (A) Regions that show greater positive (red) or negative (blue) bivariate correlations with the whole-brain contrast of Pvt connectivity compared with the average signal from the rest of the thalamus (Pvt bivariate map ∩ Pvt > Rest of the thalamus, both maps thresholded separately at *p*TFCE-FWE<0.05). **(B)** For the regions showing greater positive connectivity of the Pvt (yellow) compared to the rest of the thalamus (purple), raincloud plots display density plots with the individual Fisher *r*-to-*Z* connectivity values for each participant for a priori regions of interest. The Pvt signals are shown in yellow and the signal from the rest of the thalamus is shown in purple. From top-to-bottom subplots are displayed in descending order based on effect size as calculated by paired Cohen's d.

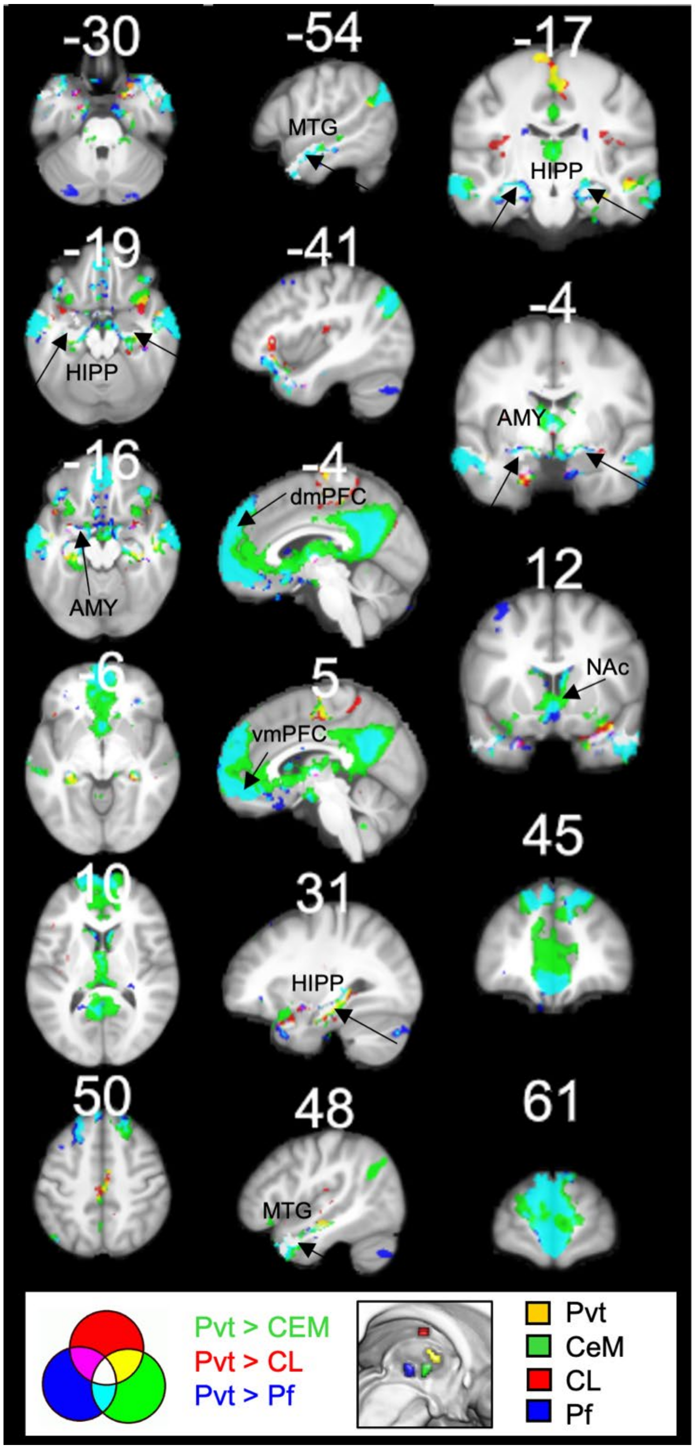

Supplementary Figure 8. Results of control analysis using small portions of the three thalamic subnuclei control seeds to closer approximate the voxel extent of the Pvt. New bilateral CeM, CL, and Pf seeds were drawn within the bounds of the original Krauth seeds (see inset depiction of seeds). The most comparable in size to the $14mm^3$ Pvt (yellow) was the smaller version of the CL ($20mm^3$) followed by the CeM ($36\ mm^3$) and Pf ($55\ mm^3$). Results depict regions that show positive Pvt bivariate FC that is greater than connectivity of the smaller versions of the control seeds: CeM (green), CL (red), or Pf subnuclei seed regions (blue) (e.g., Pvt bivariate map ∩ Pvt > CL bivariate maps both thresholded separately at *p*TFCE-FWE<0.05). Some areas showed greater connectivity compared to CL and CeM (yellow), CeM and Pf (cyan), CL and Pf (violet), or all three (white). Inset

color mixing image (lower left) access from https://colourware.wordpress.com/ (colourmixing.gif, 2013)). Black arrows and labels highlight regions shown in white that were also shown in the main text to have greater positive FC for the Pvt. Abbreviations: AMY=amygdala, dmPFC=dorso-medial PFC, HIPP=hippocampus, MTG=middle temporal gyrus, NAc=nucleus accumbens, vmPFC=ventro-medial PFC.

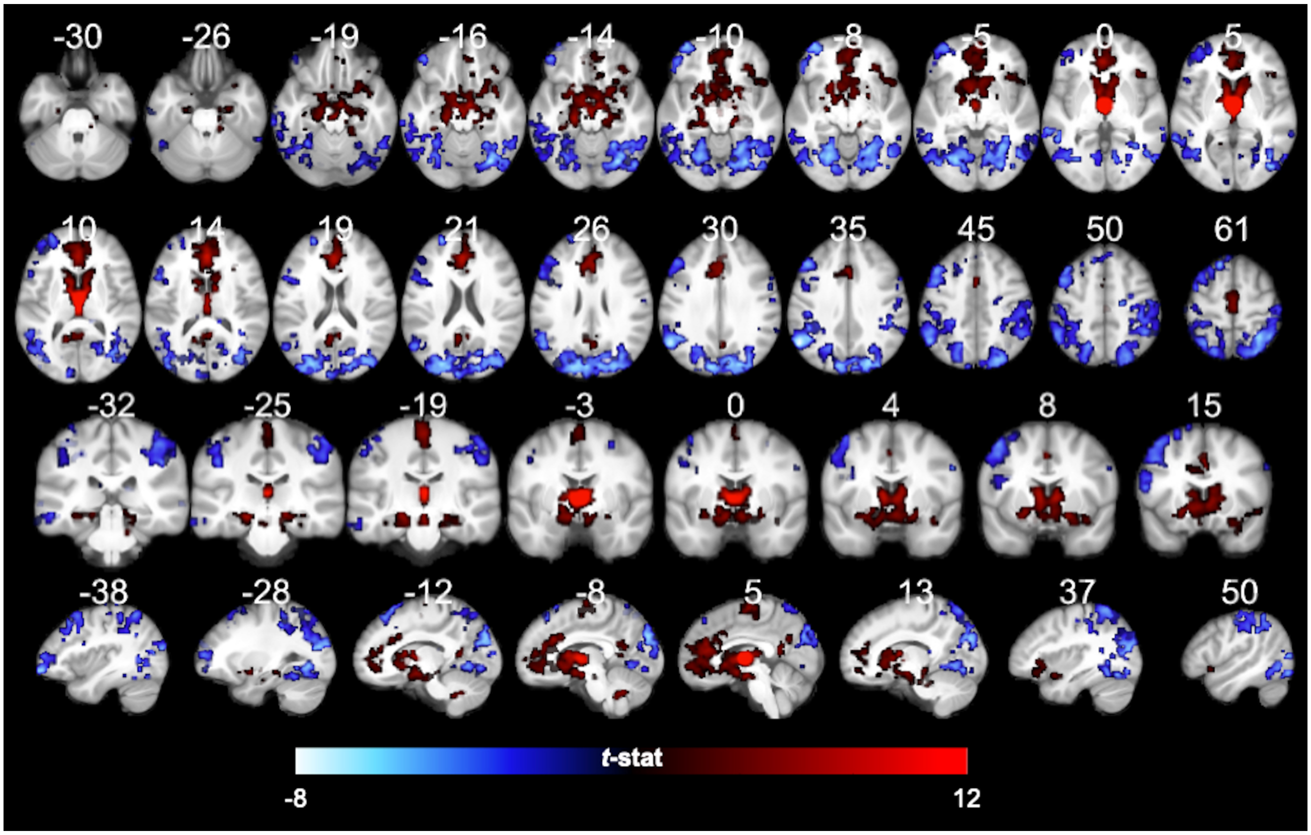

Supplementary Figure 9. 3T Pvt functional connectivity. Positive (red) and negative (blue) functional connectivity of the Pvt (key slices shown on average subject anatomical) controlling for average signal from the rest of the thalamus. Results thresholded at *p*TFCE-FWE<0.05. Compare to the 7T results in Figure 2 of the main article.

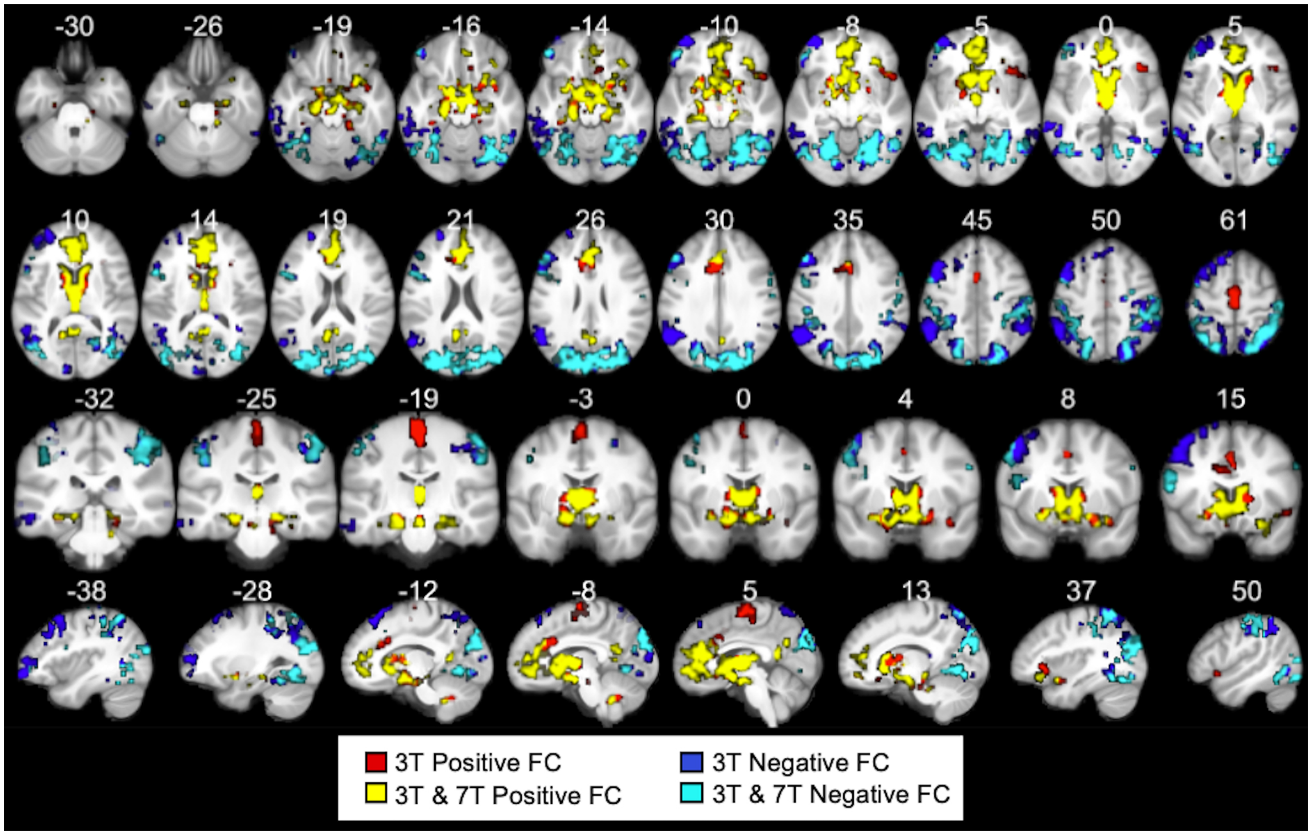

Supplementary Figure 10. 3T Pvt functional connectivity showed with overlap of the 7T results. Positive (red) and negative (blue) functional connectivity of the Pvt (key slices shown on average subject anatomical) controlling for average signal from the rest of the thalamus. Regions demarcated in yellow and cyan illustrate regions that showed positive (yellow) and negative (cyan) FC in both the 7T and 3T datasets. All results thresholded at *p*TFCE-FWE<0.05. Compare to the 7T results in Figure 2 of the main article.

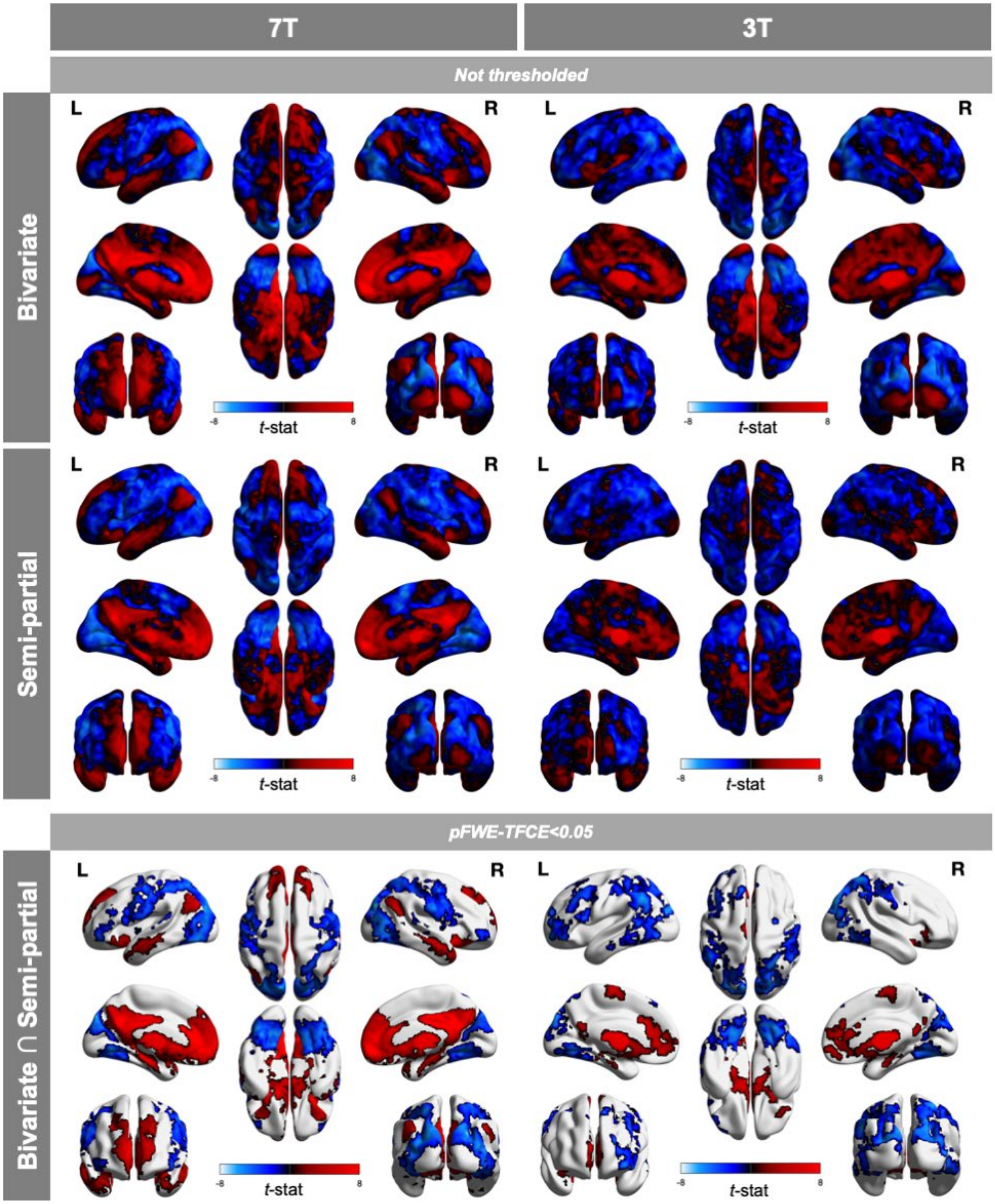

Supplementary Figure 11. Unthresholded (upper two rows) and thresholded (bottom row) 7T and and 3T data by seed-based connectivity map type (i.e., bivariate or semi-partial correlation controlling for the rest of the thalamus).
